# Recovery and analysis of ancient beetle DNA from subfossil packrat middens using high-throughput sequencing

**DOI:** 10.1038/s41598-021-91896-8

**Published:** 2021-06-16

**Authors:** Aaron D. Smith, Marcin J. Kamiński, Kojun Kanda, Andrew D. Sweet, Julio L. Betancourt, Camille A. Holmgren, Elisabeth Hempel, Federica Alberti, Michael Hofreiter

**Affiliations:** 1 Department of Entomology, Purdue University, 901 W. State Street, West Lafayette, IN 47907 USA; 2 Zoological Museum, Museum and Institute of Zoology, Polish Academy of Sciences, Wilcza 64, 00-679, Warszawa, Poland; 3 USDA Systematic Entomology Laboratory, C/O Smithsonian Institution, National Museum of Natural History, Washington, DC USA; 4 Department of Biological Sciences, Arkansas State University, State University, AR 72467 USA; 5 Science and Decisions Center, U.S. Geological Survey, Reston, VA USA; 6 Department of Geography and Planning, SUNY Buffalo State College, Buffalo, NY USA; 7 Institute for Biochemistry and Biology, University of Potsdam, Potsdam, Germany; 8 Museum Für Naturkunde, Berlin, Leibniz Institute for Evolution and Biodiversity Science, Berlin, Germany; 9 Reiss-Engelhorn-Museen, Mannheim, Germany

**Keywords:** Entomology, Phylogenetics

## Abstract

The study of ancient DNA is revolutionizing our understanding of paleo-ecology and the evolutionary history of species. Insects are essential components in many ecosystems and constitute the most diverse group of animals. Yet they are largely neglected in ancient DNA studies. We report the results of the first targeted investigation of insect ancient DNA to positively identify subfossil insects to species, which includes the recovery of endogenous content from samples as old as ~ 34,355 ybp. Potential inhibitors currently limiting widespread research on insect ancient DNA are discussed, including the lack of closely related genomic reference sequences (decreased mapping efficiency) and the need for more extensive collaborations with insect taxonomists. The advantages of insect-based studies are also highlighted, especially in the context of understanding past climate change. In this regard, insect remains from ancient packrat middens are a rich and largely uninvestigated resource for exploring paleo-ecology and species dynamics over time.

## Introduction

The study of ancient DNA (aDNA) has fascinated both researchers and the public ever since the first reports were published exploring its feasibility^1^. Progress in molecular techniques and verification protocols have enabled scientists to publish reliable aDNA datasets^2,3^, which have been employed in a variety of biological studies^4^. While the majority of available aDNA data are from mammals, including hominins, publications on birds^5^, molluscs^6^, plants^7^, fungi^8^, viruses^9^ and other organisms are also available.

Besides a few contributions in the 1990’s that are now widely accepted to represent contamination^10^, only a handful of replicable insect aDNA studies exist^11–13^. To date, the oldest (> 100,000 years) invertebrate DNA sequences were obtained from deep ice core samples from Southern Greenland^13^. However, only partial cytochrome oxidase subunit 1 (COI) sequences were recovered, and the authors were unable to assign taxa below the ordinal or familial level. The only other invertebrate aDNA study used permafrost sediment samples, ranging from ca. 10,000 to 48,000 years, and yielded partial COI and 16S sequences for different beetle species^14^. However, the estimated probabilities of assigning sequences to a given taxonomic level were low. These studies illustrate a main trend in insect aDNA studies, which have been mostly focused on demonstrating the feasibility of extracting endogenous DNA rather than investigating particular biological problems—unlike published aDNA studies on vertebrates^4^. The only available insect aDNA paper dealing with a phylogenetic question at the species level was based on 400-year-old samples^12^.

One of the main reasons why insect aDNA is not already implemented in a wide variety of biological studies is likely the relative scarcity of subfossil insect material from which DNA can be recovered. Generally, the best-preserved samples, in terms of DNA stability, originate from cold environments at high latitudes and elevations^15^. However, another, largely untapped source of well-preserved ancient specimens (including insects) are subfossilized rodent middens^9,11,16^. Ancient rodent middens are amalgamations of plant and animal remains embedded in blocks of crystallized urine preserved in aridland caves and rock shelters^17^. Distributed across arid parts of Australia (built by *Leporillus*), Central Asia (*Alticola*, *Ochotona*), North (*Neotoma*) and South America (*Abrocoma, Phyllotis*), and southern Africa (*Petromus*), middens are frequently used to investigate myriad ecological responses to environmental change over the last 50,000 years^18^. The feasibility of extracting insect aDNA out of this paleontological resource has already been shown^19^. However, the amount of sequenced DNA in these studies was insufficient for species-level identification.

Previous insect aDNA papers have been based on Sanger sequencing^11–14^ or metagenomic methodologies^16,19^. This translates directly to the above-mentioned scarcity of available sequences. However, multiple recent studies of historical materials (museum specimens) have shown the feasibility of recovering and assembling longer DNA fragments from insect specimens using high-throughput shotgun sequencing methods^20–22^. In addition, extraction and library preparation protocols for aDNA analyses are constantly being developed towards maximizing yield from older samples^3^. This theoretically provides a firm background for molecular studies using subfossilized insects, while also exploring the ability of existing aDNA extraction methods to recover sequence data from relatively small specimen fragments.

The present paper reports the successful extraction and analysis of insect mitochondrial genomes and nuclear ribosomal DNA (28S) from ancient packrat (*Neotoma*) middens (up to ~ 34,355 years before present, ybp) using single-stranded library preparation combined with high-throughput shotgun sequencing. Due to the amount of recovered data, specimen identifications were confirmed to the species level.

## Materials and methods

### Ancient samples

Studied insect subfossils originated from midden samples collected at Joshua Tree National Park in southeastern California (USA) and the eastern piedmont of Sierra Juarez in northern Baja California (Mexico) with calibrated median ages from 1615 to 34,355 ybp. As studies of plant macrofossils and pollen samples from those middens have been published, detailed technical descriptions of the investigated middens already exist^23,24^. However, a brief description is given here. From each midden a minimum of 500 g of material has been collected. Middens were first inspected and the rind was removed to rid the sample of modern contaminants. In the laboratory, middens were washed and dried. Subsequently the macrofossils were sorted and stored at room temperature in sterilized tubes. Material for radiocarbon dating (rodent fecal pellets or plant macrofossils) was pretreated at California State University, Long Beach, and measured at the W. M. Keck Carbon Cycle Accelerator Mass Spectrometry Laboratory at the University of California, Irvine. The Calib 5.0.2 Intcal04 calibration curve (Stuiver and Reimer, 1993) was used for samples < 21 ^14^C ka BP and the CalPal-2007-Hulu curve was used for samples > 21 ^14^C ka BP (www.calpal.de). Dates are reported herein as the midpoint of calibrated age ranges (Table 1).

**Table 1:** Summary of ancient samples reported in this study.

Calibrated age midpoint (ybp)	Original sample code	Origin	Available/used* body fragments	DNA extract concentration (ng/μl)	Library concentration (ng/μl)	Amount used for lib. prep	Total # of recovered reads	# of aligned genomic^a^ reads	# of aligned mitochondrial^b^ reads
1615	53^23^	Joshua Tree National Park (JTNP) (Jumbo Rock)	Pronotum, coxa + femur + tibia*	0.558	10.9	Whole extract (20 µl)	2,252,684	3511 (0.156%)	6777
2035	74^23^	JTNP (Jumbo Rocks North)	Femur + tibia*	1.06	16.2	11 µl	2,262,666	3973 (0.176%)	12,073
8480	80B^23^	JTNP (Hidden Valley)	Femur + tibia*	NM	12.9	Whole extract (20 µl)	2,386,019	712 (0.030%)	601
16,610	41B^24^	Sierra Juarez (Guadalupe Canyon)	Femur + tibia*	0.548	19.3	Whole extract (20 µl)	–	–	–
34,355	60B^23^	JTNP (Indian Cove)	Abdominal ventrites, pronotum, femur + tibia* (3 sets)	1.99	10.5		2,471,490	133 (0.005%)	45
–	Ex Blk1	–	–	NM	3.34	Whole extract (20 µl)	473,099	0	1
–	Libr Blk	–	–	–	2.88	–	292,658	0	–

*Body part used for extraction; + intact body parts; NM not measurable; Ex and Libr Blk extraction and library blanks; ^a^Endogenous genomic DNA content measured as the total number of recovered reads mapped to the *Tribolium castaneum* genome; ^b^Number of reads mapped to the reference mitogenome of *Philolithus actuosus.*

Prior to this study, the subfossil arthropod samples were already separated and stored at room temperature in sterilized tubes^23,24^. These arthropod fragments were examined in their original vials before selecting five subfossils of Asidini (darkling beetles) for potential DNA extraction (Table 1). The samples were then examined separately outside the original vials to confirm identifications at Northern Arizona University (NAU), in a room without other arthropod specimens, using a sterilized microscope, nitrile gloves, and previously unused and sterilized forceps and disposable petri dishes. The samples, primarily legs, were identified as belonging to *Philolithus actuosus* based on the presence of several distinct morphological characters, particularly the lack of an apical protibial spine, the presence of crenulations along the inner tibial margins, and the rugosity and punctation on femora. These characters are sufficient to both identify *Philolithus* from other co-occurring genera, and to separate *P. actuosus* from *P. carinatus*, a similar species known from southern California. Post-examination at NAU, samples were resealed in sterile 1.5 ml microtubes and sent to the University of Potsdam for extractions, where they were processed in the ancient DNA laboratory of the Evolutionary Adaptive Genomics research group. Prior to this, no samples representing the family Tenebrionidae were handled by this facility, while the distribution of the studied species is restricted to the southern Nearctic Realm, which precludes any forms of accidental contamination at this step.

### Extraction, library preparation and sequencing

*Ancient midden taxa*. DNA was extracted by combining the protocols of Dabney et al.^2^ and Rohland et al.^25^ with a modified extraction buffer from Taron et al.^26^. Specifically, tibiae and femora were cut longitudinally and later incubated in 1 mL of extraction buffer (5 M GuSCN, 25 mM NaCl, 50 mM Tris, 20 mM EDTA, 1% Tween-20, 1% beta-Mercaptoethanol) for 20 h at 37 °C on a rotating wheel. Subsequently, centrifugation was performed to pellet the remaining tissue, and the supernatant was combined with 13 mL of binding buffer (5 M guanidine hydrochloride, 40% isopropanol, 0.05% Tween-20, and 90 mM sodium acetate). Purification was performed on a Zymo-Spin V Column reservoir combined with a Qiagen MinElute column. Two washing steps were performed using PE Buffer (Qiagen), followed by a drying spin for 1 min at 13,000 rpm. DNA was eluted twice, each using 12.5 µL TET buffer (10 mM Tris–HCl, 1 mM EDTA, 0.05% Tween-20), using a 10 min incubation time. Two blanks, one handled first and the other last, were included during the extraction process (Table 1). They were later merged into a single tube and processed along with the samples after determining that DNA concentration was too low in both to be measurable. An additional blank was also included for the library preparation steps.

Illumina sequencing libraries were prepared using a protocol based on single-stranded DNA^3^. The extracts were quantified using a Qubit 2.0 instrument (Fisher) with the dsDNA HS Assay kit (Table 1). The input volume for library preparation was adjusted to 13 ng total input DNA to maximize the efficiency of the single-stranded ligation reaction^3^. Input DNA was first treated with uracil-DNA glycosylase and endonuclease VIII to remove uracils, which are produced from the deamination of cytosines in ancient or degraded DNA^27–29^. In this process, the input volume extractions were added to a mix containing 11 μl nuclease free water, 8 μl Circligase buffer II (10×), 4 μl MnCl2 (50 mM), 0.5 μl Endonuclease VIII (10 U μl − 1) and 0.5 Afu UDG (2 U μl − 1) per sample^30^. This solution was incubated at 37 °C for 1 h.

Libraries were PCR amplified and indexed by adding 8 μl Accuprime Pfx reaction mix (10×), 3.2 μl P7 indexing primer (10 μM) 3.2 μl P5 indexing primer (10 μM), 0.8 μl Accuprime Pfx polymerase (2.5 U μl − 1) and 44.8 μl nuclease free water followed by a selected number of PCR cycles, involving denaturation for 15 s at 95 °C, annealing for 30 s at 60 °C and primer extension for 1 min at 68 °C. The appropriate numbers of cycles were established using qPCR (PikoReal Real-Time PCR system, Thermo Fisher Scientific) analysis of the unamplified libraries to identify the cycle number corresponding to the point of inflection of the qPCR amplification curve, correcting for differing reaction volume and template amount in the subsequent library amplification PCR. After amplification, the indexed libraries were quantified using a Qubit with the dsDNA HS Assay kit (Table 1). For each library, ~ 2.25–2.47 million 75 bp PE reads were sequenced on an Illumina NextSeq 500 sequencing platform at the University of Potsdam, Germany.

*Museum and modern taxa* The identities of the ancient insect fragments were tested in a phylogenetic context. The DNA alignment for phylogenetic inference included sequences from aDNA samples, two museum specimens of *Philolithus actuosus*—putative conspecifics to the subfossil fragments, and 15 additional Asidini, i.e., 13 species from the Southwest United States and two outgroup species from the Southern African genus *Machla* (Supplement 3), which were also in reference-based assemblies for the ancient samples.

DNA from both museum specimens of *Philolithus actuosus* was extracted under a UV-sterilized laminar flow hood with dedicated equipment using QIAamp DNA Micro kits (Qiagen) following the manufacturer’s protocol with the addition of carrier RNA. DNA was extracted from head capsules, without grinding the cuticle, to minimize damage to specimens. Libraries were constructed using 30 to 50 ng DNA with NEBNext® Ultra™ II DNA Library Prep Kits (New England Biolabs) following the manufacturer’s protocols. Libraries were sequenced on an Illumina MiSeq maintained by the Environmental Genetics and Genomics Laboratory (Northern Arizona University, Flagstaff AZ), using 75 bp paired end runs.

Sequence data from other Asidini genera (*Machla*—TB17124, *Pelecyphorus*—TB21116A, and *Stenomorpha*—TB15559C) and one *Philolithus aegrotus* specimen (TB15569) were obtained using a low-coverage genome sequencing approach. DNA was extracted from the whole body to maximize DNA yield using DNeasy Blood and Tissue Kits (Qiagen) following the manufacturer’s protocols. Cuticle was not ground to minimize damage to the specimens. Library preparations were performed with NEBNext® Ultra™ II DNA Library Prep Kits (New England Biolabs), following the manufacturer’s protocols, on 300–1000 ug of DNA per specimen. Libraries were sequenced at the University of Arizona’s Genomic and Technology Core Facility (UAGC) on an Illumina Hi-Seq using 150 bp paired end runs.

Molecular data for the remaining *Philolithus* specimens, excluding one *Philolithus aegrotus* (TB15569), were obtained using Sanger sequencing for mitochondrial (COI, COII, and 12S) and nuclear (28S) loci. DNA was extracted from heads using DNeasy Blood and Tissue Kits (Qiagen) following the manufacturer’s protocols. PCR reactions were performed using primer pairs and thermocycler programs described in Kanda et al.^20^ and Kamiński et al.^22^. PCR products were cleaned, quantified, and sequenced at the University of Arizona’s Genomic and Technology Core Facility (UAGC) using a 3730 XL Applied Biosystems automatic sequencer. Chromatograms were assembled using Phred v. 0.020425.c^31^ and Phrap v. 0.990319^32^, implemented in Mesquite’s Chromaseq v. 1.5 package^33^ with manual inspection.

### Mitochondrial genome assembly

Before assembly, the modern, museum, and ancient libraries were trimmed using Trimmomatic v.0.36^34^. For modern and museum libraries, trimming removed adapters, trailing bases below a PHRED quality of 3, sliding windows (4 bp) below an average PHRED quality of 15, and any reads < 20 bp after trimming. Ancient libraries were trimmed by removing adapters and reads < 30 bp. All trimmed libraries were then assessed using FastQC v.0.11.7 (Babraham Bioinformatics). The mitochondrial genomes of modern samples and the two most recent ancient samples (~ 1615 ybp and ~ 2035 ybp) were assembled de novo using the multi-cell mode in SPAdes v.3.13.0^35^. Assembled mitochondrial scaffolds were identified using BLAST searches against the published mitochondrial genome from *Asbolus verrucosus* (NC_027256). Mitochondrial genomes of the two museum specimens of *Philolithus actuosus* were then assembled by mapping reads to the modern *P. aegrotus* mitochondrial genome in MITObim v.1.8^36^. Finally, mitochondrial genomes for the ancient samples were also assembled by mapping reads to the mitochondrial genome of the *P. actuosus* Joshua Tree specimen using the *aln* algorithm in BWA v.0.7.17 both with (default) and without seeding^37^. The two control blanks were also mapped to this reference. SAMtools v.1.8^38^ was used to remove duplicate reads, sort reads, remove unmapped reads, and remove reads with Mapping Quality scores < 30. BCFtools^39^ was then used to generate pileup files and consensus FASTA sequences for each filtered BAM file. To ensure reference mapped mitogenome sequences of ancient samples were not biased by the reference, each ancient library was also mapped to four additional mitogenomes from modern samples (Fig. 2a): *P. aegrotus, Pelecyphorus contortus, Stenomorpha consobrina,* and *Machla setosa*. Finally, the filtered BAM files were run against the *P. actuosus* reference in mapDamage v.2.0^40^ to assess fragment lengths, breakpoints, and deamination (Supplement 6).

All mitochondrial genome assemblies (modern, museum, and ancient) were then annotated using the MITOS2 web server (http://mitos2.bioinf.uni-leipzig.de/index.py)^41^ and manual curation following the workflow outlined in Cameron^42^. Average sequencing depth for each mitochondrial genome assembly was calculated using SAMtools and per-base coverage was calculated using BEDTools v.2.29.0^43^.

### 28S assembly and nuclear genome read-mapping

28S sequences were assembled from the Illumina libraries of modern, museum, and ancient samples using a read-mapping approach in BWA. Each modern sample was mapped to a 28S sequence of the same genus generated from PCR and Sanger sequencing, while museum samples were mapped to the 28S sequence assembled for *P. aegrotus*. To ensure that mapped sequences of ancient samples were not biased by the reference, each ancient library was mapped to 28S sequences from *P. actuosus*, *P. aegrotus*, *Pelecyphorus contortus*, *Stenomorpha consobrina*, and *Machla setosa* (Fig. 2b). Resulting BAM files were sorted and filtered in SAMtools, and consensus sequences were generated using BCFTools.

To assess nuclear coverage of the ancient samples, Illumina libraries were mapped to the nuclear genome of *Tribolium castaneum*^44^ in BWA (again using both seed and seedless parameters) with the *aln* algorithm. SAMtools and BEDTools were then used to remove duplicate reads, sort reads, remove unmapped reads, filter (MQ < 30), and calculate sequencing depth and proportions of reads that mapped to the reference.

### Phylogenetic analysis

The resulting sequences were aligned by locus in MAFFT V7.402^45^ and then concatenated in Mesquite 3.61^33^ into a single matrix (13,926 bp) for phylogenetic analyses. Data partitions and models of sequence evolution were assessed with Partitionfinder v. 2.1.1 as implemented on CIPRES^46^, with the concatenated dataset initially partitioned by gene and codon position (for protein coding genes) using the greedy searches and the Bayesian information criteria (BIC). The best-fit partitioning scheme is presented in Supplement 5.

Phylogenetic analyses were conducted on CIPRES using MrBayes v. 3.2.7a^47^ and IQ-Tree v. 1.6.10^48^. Bayesian analyses were performed with two independent runs, each with four chains sampled every 1000 generations after a burnin of 25%, with convergence checked using the average standard deviation of split frequencies and in Tracer v. 1.7.1^49^. Posterior probability (PP) values were used to assess branch support for the resulting Bayesian consensus topology. Maximum likelihood (ML) analyses in IQ-Tree were run with edge-unlinked partition models (− spp), with partitions and models reassessed in IQ-Tree prior to phylogenetic analysis. Branch support was estimated in IQ-tree using 20,000 ultrafast bootstrap replicates^50^, with the ‘bnni’ approach to reduce the risk of overestimating support values.

## Results

### aDNA sequence assembly

To estimate the overall endogenous DNA content of the studied ancient samples (Table 1), recovered sequence data were mapped to the only available Tenebrionidae nuclear genome assembly—that from *Tribolium castaneum*^44^—a distantly related species from the subfamily Tenebrioninae, that likely diverged from Pimeliinae (the subfamily Asidini belongs to) ~ 145 million years ago^51^. As such, the presence of endogenous beetle DNA should be interpreted qualitatively, i.e., beetle DNA was present, rather than quantitatively (though relative quantities are shown)^52^. The endogenous content recovered from the samples varied between 0.005–0.176% (Table 1). For phylogenetic purposes, a portion of the nuclear ribosomal gene 28S sequence (D1–D3 region) was also recovered from the samples dating back to ~ 34,355 ybp as well as mitogenomic data.

**Figure 1:**
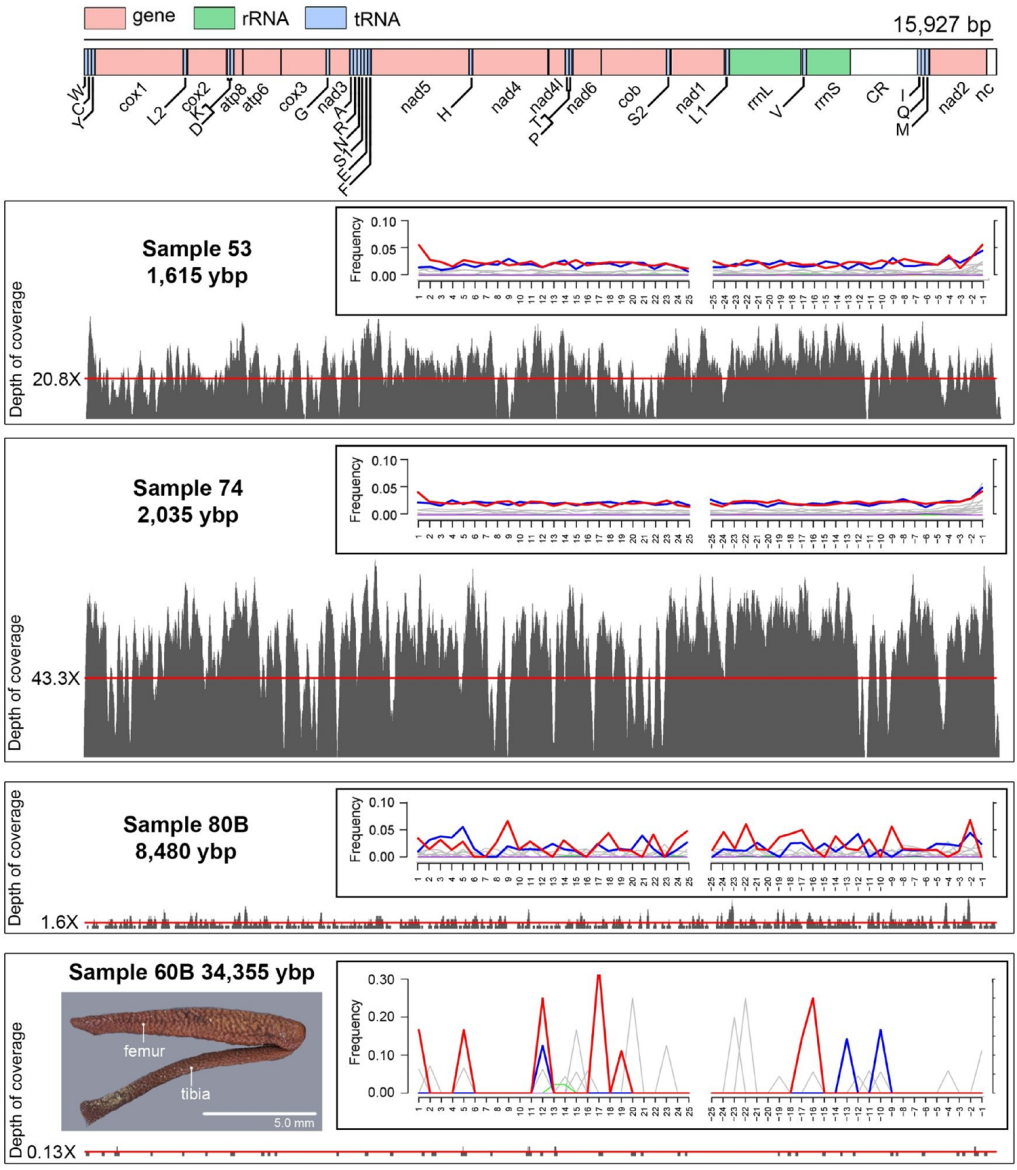
Gene arrangement and read depth along reconstructed ancient mitochondrial genomes. All aDNA reads were mapped in BWA v.0.7.17 to the *Philolithus actuosus* TB20958 (Tenebrionidae: Asidini) mitogenome. Asterisk indicates samples containing > 1% ambiguous sequences from lack of coverage. Red lines mark average coverage. White fields represent control (CR) and other non-coding regions (nc). Color codes: Red: C to T substitutions; Blue: G to A substitutions; Grey: All other substitutions; Orange: Soft-clipped bases; Green: Deletions relative to the reference; Purple: Insertions relative to the reference. Subfossil fragments (*Philolithus actuosus*) used in the extraction process were illustrated for 34,355 ybp sample.

Mitochondrial genomes were assembled and annotated for radiocarbon dated samples from ~ 1615 and ~ 2035 ybp (Fig. 1). The assembled mitochondrial genomes were 16,076 and 16,114 bp long, respectively. The average read depth was 21-fold for the ~ 1615, and 43-fold for the ~ 2035 ybp samples (Fig. 1). The nucleotide composition of the recovered mitogenomes were heavily AT-biased (average AT content = 72.8%). The recovered gene arrangement for *Philolithus actuosus* is consistent with those reported for other darkling beetle species^53^, with the addition of an intergenic non-coding region following *nad2* (Fig. 1). This gene arrangement is also consistent with the inferred ancestral mitochondrial gene order in insects^54^. Partial mitochondrial genomes were recovered for the samples dated at ~ 8480 and ~ 34,355 ybp (Fig. 1). The average read depth for those older samples was low. For the ~ 8480 ybp sample, 72.8% of the aligned mitochondrial bases present in the matrix were covered by at least one read, with an average read coverage across the mitogenome of 1.6X. For the ~ 34,355 ybp sample, 12.3% of the aligned mitochondrial bases were covered by at least one read, with an average read coverage across the mitogenome of 0.13X. For the ~ 16,610 ybp sample no unambiguous sequences were recovered (Fig. 1).

**Figure 2:**
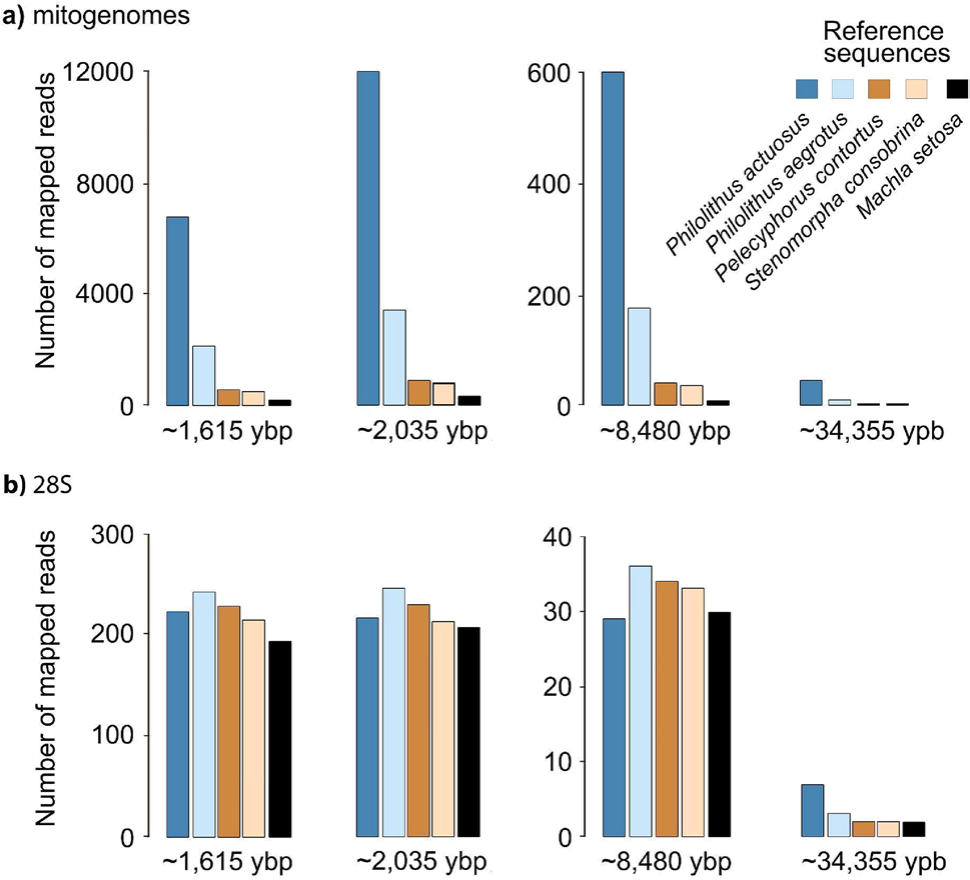
Comparison of the number of successfully recovered mitochondrial (**a**) and nuclear (**b**) reads from the analyzed libraries representing ancient samples mapped to different reference sequences. Subsequent reference sequences represent species increasingly less related to the analyzed ancient species based on morphological traits.

In the case of all analyzed ancient DNA libraries, the highest number of mitochondrial reads mapped to sequences originating from *Philolithus actuosus* (Fig. 2a), which based on morphological traits was suspected to constitute the closest reference*.* The number of mapped reads drops by over 60% in more distantly related references, i.e. *Philolithus aegrotus, Pelecyphorus contortus, Stenomorpha consobrina,* and *Machla setosa*. This tendency was not observed in all 28S sequences, where for ~ 1615 ybp, ~ 8480 ybp, and ~ 34,355 ybp samples, the number of recovered reads remained on a similar level regardless of the reference sequences used (Fig. 2b). This is likely due to the relatively conserved nature of 28S in comparison to the faster evolving mitogenome^55^. From the sequenced beetle extraction blanks, a single read of 52 bp mapped to the tRNA-Trp when using *Philolithus actuosus* or *Philolithus aegrotus* as the reference sequence. A GenBank search (blastn) placed the sequence as potentially beetle DNA (Scraptiidae: *Anaspis*, max. score 81.5), but it is unclear if this was from the extractions or environmental DNA. The one Pimeliinae mitogenome in GenBank (from *Asbolus verrucosus*) was not recovered in the top 100 sequences producing significant alignments (max scores 81.5–60.8). No other reads from the extraction blanks, nor any from the library blank mapped to any of the references.

The results of the MapDamage analysis of the mapped mitochondrial reads are presented on Fig. 1. Provided patterns concern the UDG-treated DNA, and therefore should be interpreted with caution^28^. Furthermore, due to scarcity of obtained reads, the MapDamage profiles recovered for older samples (i.e., ~ 8480 and ~ 34,355 ybp) are not alone conclusive. On the other hand, the mapped reads for the ~ 1615 and ~ 2035 ybp samples show somewhat elevated levels of C → T substitutions relative to the reference genome at their terminal ends, which is expected for ancient samples^56^. At the same time, it has to be noticed that these samples are not old enough to expect massive deamination^57^.

### Phylogenetic placement of ancient specimens

To test the reliability of the DNA recovery process, phylogenetic analyses were performed based on a matrix composed of 15 mitochondrial loci (16S, 12S, atp6, atp8, cob, COI-III, NAD1-6) and the D1–D3 region of nuclear ribosomal 28S, for a total of 13,926 bp. Selected species representing Asidini were incorporated in order to identify the ancient samples to species level, with ancient DNA reads mapped to multiple taxa and the mitochondrial genes for the two most recent samples assembled de novo (Supplement 1). The greatest read coverages for the ancient samples were recovered when *Philolithus actuosus* was used as the reference taxon (Supplement 2). All ancient samples from which DNA was recovered were placed as *Philolithus actuosus* with high probability (PP: 1.00 and BS: 97; Fig. 3). This species currently occurs in and around Joshua Tree National Park in southeastern California and this study indicates that it has likely persisted in the region for some time.

**Figure 3:**
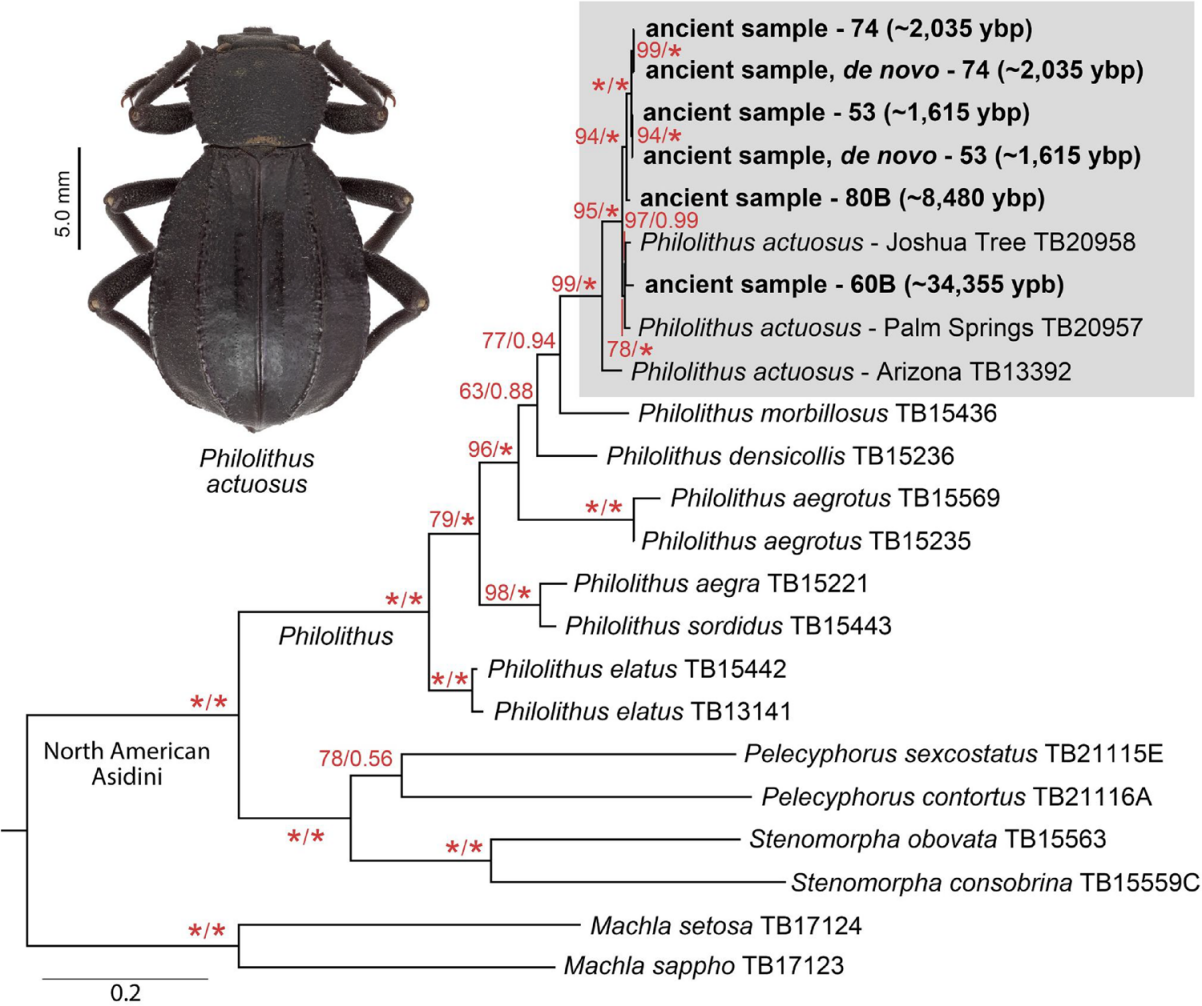
Phylogenetic placement of recovered ancient samples. Maximum likelihood tree generated by analysis of a concatenated dataset of 13,926 bp (16 s, 28 s, 12 s, atp6, atp8, cob, COI-III, NAD1-6) in IQ-Tree with ancient midden samples assembled de novo or using *Philolithus actuosus* (TB20958) as the reference. Total number of bp used for each taxa is listed in Supplemental Table 1. Posterior probabilities (PP) from Bayesian analyses are displayed on the right, bootstrap values from IQ-Tree on the left. An asterisk indicates either PP of 1.0 or bootstrap value of 100%.

Additional phylogenetic analyses were conducted with ancient samples mapped to multiple references (*Philolithus actuosus*, *Philolithus aegrotus*, *Pelecyphorus contortus*, *Stenomorpha consobrina*, and *Machla setosa*), and using different read mapping parameters, to explore potential reference bias (Supplements 3, 4) and sequence recovery rates (Fig. 2). Minor differences between mapped sequences using seed versus seedless parameters (six or less bp changes) occurred in a few loci, but did not affect placements in single-loci maximum likelihood topologies; hence, assemblies using seed parameters were used for subsequent analyses. While a degree of reference bias was evident in the phylogenetic analyses, it had only a slight impact on the overall placement of the ancient samples. The majority of ancient libraries mapped to different references were still recovered as *Philolithus actuosus*, with relatively small genetic differences between each other. Five alternate assemblies were placed differently when all references were used. The ~ 8480 ybp library mapped to *Pelecyphorus contortus* and *Stenomorpha consobrina* were both recovered within *Philolithus*, but not with other *Philolithus actuosus* sequences. The ~ 1615 ybp, ~ 2035 ybp, ~ 8485 ybp libraries mapped to *Machla setosa* were all recovered outside of the genus *Philolithus* (Supplement 4)*.* However, in all of these cases the number of mapped reads and the total number of base pairs present in the matrix (i.e., most reads mapped to conserved regions) were extremely low when compared to phylogenetically closer references (Fig. 2a, Supplement 2). Hence, the phylogenetic reliability of the resulting assembled sequences is questionable. For example, only 12 reads were recovered from the ~ 8480 ybp sample mapped to *Machla setosa* (Fig. 2a, Supplement 2). Only one of these reads mapped to a protein coding gene (NAD4), and was identical to that portion of the reference. The remaining 11 reads mapped to tRNAs or rRNAs, which could be mismappings or mappings to highly conserved regions. Bayesian phylogenetic analyses using alternate references (i.e., any reference other than *P. actuosus*) uniformly failed to converge when run for 20 million generations.

## Discussion

### Challenges of ancient DNA studies using insects

Compared to the number of recent publications dealing with animal aDNA^2,4–6^, the scarcity of insect studies is striking. To our knowledge, this is the first targeted study on insect aDNA using high-throughput sequencing, despite the widespread availability of next-generation sequencing technologies over the past ~ 15 years. We assume that the lack of such studies is partially explained by difficulties with reliably identifying ancient material, which makes insects an unappealing research subject for some scientists. The species diversity within insect communities in any given area is also often considerably higher than that of other animal groups. For example, within the borders of Białowieża Primaeval Forest, Europe's largest surviving primeval forest, 59 mammal species (including rodents and bats) have been recorded^58^, while around 9,600 insect species are known to inhabit the area^59^. In one instance, the coexistence of 20 relatively closely related and morphologically convergent (difficult to distinguish) coprophagous scarab beetles was reported from just a small fragment (200 m^2^) of that ecosystem^60^. As many insect groups lack comprehensive, well-illustrated taxonomic contributions, reliable species identifications, especially for damaged subfossil specimens, can often only be made by taxonomists specializing in a specific group (generally at the ordinal or family level) or geographic region, which generally imposes a need for cooperation from multiple taxon experts to solve a particular problem in a given area.

On the other hand, insect identification based solely on molecular data is also problematic. Compared to vertebrates (specifically birds and mammals), many insect groups lack published phylogenetic hypotheses and/or molecular data to explore the placement of ancient taxa. For example, prior to this study there was a single publication available on the phylogeny of North American Asidini^61^—the tribe containing our analyzed ancient specimens, while other geographic components of this tribe (especially African and South American taxa) have yet to be put within a phylogenetic framework, with no sequences currently available in GenBank. To address this limitation in the context of the current study, we sequenced the mitogenomes of seven additional Asidini species from modern samples and two museum specimens (collected ~ 54 ybp) of *Philolithus actuosus* from the geographic region of interest (Fig. 3). Globally, the scarcity of reference sequences prevents molecular species-level identification, which explains why most of the available insect aDNA studies were limited to higher classification levels^11,13^. Furthermore, the lack of available insect genomes in comparison to their diversity complicates genome reconstruction^4^, as mapping efficiency drops rapidly with increasing phylogenetic distance^52,62^. Taking this into account, we assume that the nuclear endogenous content presented here for the analyzed ancient samples (Table 1) is likely an underestimate, given that the subfamily containing *Tribolium castaneum* (Tenebrioninae), the only available reference genome for Tenebrionidae, is estimated to have diverged from Pimeliinae, the subfamily containing *Philolithus*, ~ 145 million years ago^51^. This illustrates the lack of genomic resources available for entomological studies (the order Coleoptera contains ~ 450,000 described species with five published genomes) when compared to the existing publicly available resources for the study of mammals (~ 6500 described species with over 119 published genomes). It also highlights the fact that in order to conduct aDNA-based research within the majority of insect groups, preliminary genomic sequencing and phylogenetic efforts on modern taxa are first needed to produce the required resources for aDNA studies.

In order to avoid some of the above mentioned restrictions, alternative assembly approaches can be considered [e.g.^63,64^]. Data analyzed here suggest that the de novo assembly approach can be used to potentially accelerate ancient DNA studies on at least some groups of insects, as the sequences obtained by using this method were almost fully convergent with the ones derived from the reference-based technique using a museum specimen from the same species as the samples (Fig. 3). The advantage of de novo assembly for aDNA samples has already been widely postulated in many similar cases when closely related reference genomes were not available^30^. In addition, de novo assemblies can be used to identify longer deletions or genomic rearrangements^65^. However, due to the fragmentation of ancient DNA, de novo assembly is not always feasible, as resulting assembly quality strongly depends on sequencing coverage, read length and its accuracy^66^. As a consequence, especially in cases of poorly investigated groups, a variety of different approaches should be implemented to assess the accuracy of molecular identifications. In this study, de novo mitogenome assemblies were produced for the two most recent aDNA samples (~ 1615 ybp and ~ 2035 ybp) to compare the accuracy of reference based assemblies using modern species. However, de novo assemblies could not be made for the older samples and identifying midden fragments to species is often not possible based on morphology.

### Packrat middens: a reliable source of subfossilized insects

Although the recovered MapDamage profiles are not alone conclusive to validate the authenticity of the obtained sequences, especially in the case of two older samples where not enough reads were recovered (Fig. 1), it has to be noted that, due to the nature and specific processing of the midden samples, the risk of contamination has been largely minimized. Namely, the midden structure, due to its compact/fossilised nature, does not allow modern organisms to penetrate it and therefore be subsequently incorporated within the deeper layers of its matrix^67^. As stated before, the outer layer of the investigated middens has been removed prior to other laboratory procedures preventing potential inclusion of more recent subfossilizations within the sampled layers^23,24^. Furthermore, the material was initially sorted in a facility in which no modern samples of darkling beetles were being investigated. The identification process was also carried in isolation using sterilized laboratory equipment. Finally, the processed ancient samples represent the first beetle samples analysed in the ancient DNA laboratory of the Evolutionary Adaptive Genomics research group in Potsdam, Germany. Given the Nearctic distribution of the flightless *Philolithus actuosus*, contamination during the extraction or library preparation procedures seems unlikely.

For this project, all samples were first identified from previously studied middens^23,24^ to the genus *Philolithus*, primarily based on the morphology of the legs, which were also used for extractions. All of the samples that yielded endogenous beetle DNA were from Joshua Tree National Park (JTNP), and identified as *Philolithus actuosus* based on both morphology and sequence data. While several additional *Philolithus* species have been described from central California^61^, all are considered synonyms of *P. actuosus* in an upcoming revision of the genus. *Philolithus* species are fairly ubiquitous flightless detritivores in western North American deserts, with *P. actuosus* being one of the largest (length ~ 15–35 mm) macro-invertebrates in central and southern California. The species’ persistence or frequent repatriation within JTNP adds further support to the evidence from vegetation data that the region has been relatively stable and xeric over the last ~ 34,000 years^23^.

Sequence data from the Sierra Juarez mountain range (Baja California, Mexico) fragments (~ 16,610 ybp) could not be assembled to any of the Tenebrionidae references, despite being processed and stored using the same protocols as the JTNP midden samples. This may be due to the stochastic history of packrat middens or could indicate the need for updated protocols for sample preservation and processing to increase endogenous DNA yield. In contrast to bone, systematic studies trying to optimize DNA extraction from subfossil insect remains have yet to be undertaken. We assume that insect aDNA yield can be further increased by tailoring methods to extract subfossils from the midden matrix as well as optimizing DNA extraction buffers for insect remains. Current protocols include rehydration steps that may negatively impact aDNA quality. Furthermore, because all of the subfossils analyzed were primarily intended for morphological studies, they were stored at room temperature for over a decade^23,24^, which has also been argued to negatively affect ancient DNA yields, at least from bone^68^. Future insect aDNA studies could address these potential methodological shortcomings, for example by bleaching the samples^69^, breaking down the midden matrix in ethanol or directly in the extraction buffer, and storing specimens at lower temperatures. We predict that the optimization of extraction methods for subfossil insects, and potentially the application of hybridization capture techniques, will substantially increase the endogenous DNA content and sequence complexity of aDNA libraries, allowing for more comprehensive recovery of nuclear DNA, as short nuclear ribosomal DNA (28S) fragments were already reported here^24^.

### Utility and prospects of ancient DNA studies on insects

Insects are ubiquitous and play important roles in ecosystems, which makes them an interesting subject for various types of ecological investigations, including ones based on aDNA. Specifically, insects have invaded every niche, except the oceanic benthic zone^70^. They create the biological foundation for all terrestrial ecosystems as they pollinate plants (~ 85% of angiosperms are pollinated by insects), disperse seeds^71^, cycle nutrients, maintain soil structure and fertility^72^, control populations of other organisms, and serve as a food source for other taxa^73^, as well as producing byproducts such as honeydew. Compared to large mammals—the group most commonly used in aDNA studies—many insect species have short generation times and high reproductive rates, which allows them to more rapidly respond to climate change, especially temperature fluctuations^74^. Ample evidence shows that insects are one of the first groups to respond to ongoing climate change^75^. For example, a study on butterflies in the United Kingdom showed that many species failed to track recent climate warming due to a lack of suitable habitat^76^. Furthermore, insects were shown to be reliable indicators of past climatic changes in the Quaternary, providing original insights on the topic^77^. In light of aDNA based population studies, it is important to note that contrary to large mammals^78^, ancient populations of insects were likely to be less influenced by human activity in the pre-Anthropocene period. Therefore, reconstruction of their history can provide reliable reference for distinctions between natural and human-caused evolutionary bottlenecks observed in other phylogenetic groups. It needs to be noted that this potential is almost entirely unexploited, as only a handful of studies using Bayesian skyline plots on insects exist^79^. Lastly, given the complicated life histories of many insect groups (particularly Holometabola), a single insect species can potentially provide insights on more than one ecosystem, as larvae and adults often inhabit different environments, e.g. soil dwelling larvae and epigeic imagines as in darkling beetles.

In the context of insect aDNA-based climatic and population studies, darkling beetles (Tenebrionidae) are a promising group for future research, as they are extremely abundant in midden samples^80^ and, at least in some cases, are identifiable to species based on small body fragments, such as legs, pronota or elytra (Fig. 1). These features could allow for more detailed population studies of specific species that incorporate aDNA^79,81^. On the larger scale, the family includes ~ 20,000, mostly xerophilous and apterous (limited dispersal abilities) species distributed globally^51^, which makes it a suitable group for climate change studies. Moreover, ongoing phylogenetic and phylogenomic studies on the family (e.g.^20,22,61^) are constantly improving our understanding of the group’s evolutionary history and providing new reference sequences for more detailed aDNA investigations.

In conclusion, this study pictures rodent middens as a rich and unexplored source of well-preserved insect aDNA from arid parts of the globe that can be used for various climatic and/or biological studies. The presence of endogenous beetle aDNA was recorded in samples as old as ~ 34,355 ybp (Fig. 1), in quantities sufficient for inclusion in phylogenetic analyses with both modern and museum specimens (Fig. 3). This potentially enables the incorporation of aDNA data, including that generated in this study, in more detailed population studies, such as single nucleotide polymorphisms (SNPs) analyses^81^.

## Supplementary Information

Supplementary Information 1.

Supplementary Information 2.

Supplementary Information 3.

Supplementary Information 4.

Supplementary Information 5.

Supplementary Information 6.

## Data Availability

All newly generated sequences were submitted to NCBI GenBank (Accession numbers MZ313233-MZ313247, MZ313469-MZ313476, MZ322320-MZ322327, MZ342776- MZ342786, MZ351464-MZ351487), while raw sequencing reads are available from the Sequence Read Archive (Accession numbers SRR14723036-SRR14723047).
