# Supplementary material for: Recovery and analysis of ancient beetle DNA from subfossil packrat middens using high-throughput sequencing
Source: Sci Rep. 2021 Jun 16;11:12635. doi: 10.1038/s41598-021-91896-8 (PMC8209150; doi:10.1038/s41598-021-91896-8)

**Supplement**

**Supplement 2**. Phylogenetic placement of the analyzed ancient samples assembled to different reference sequences. Maximum likelihood tree generated by analysis of a concatenated dataset of 13,926 bp (16s, 28s, 12s, atp6, atp8, cob, COI-III, NAD1-6) in IQ-Tree with displayed bootstrap values.
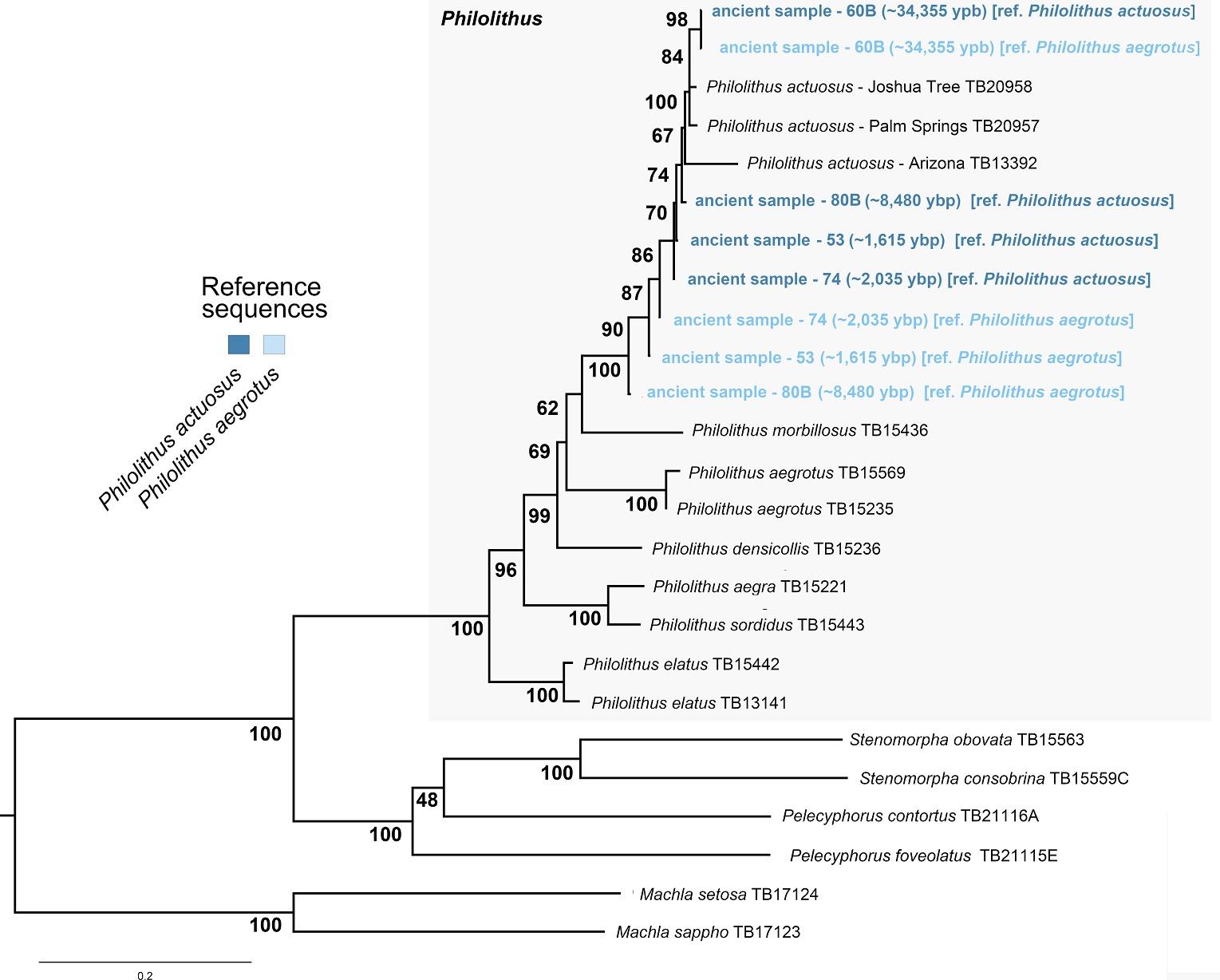

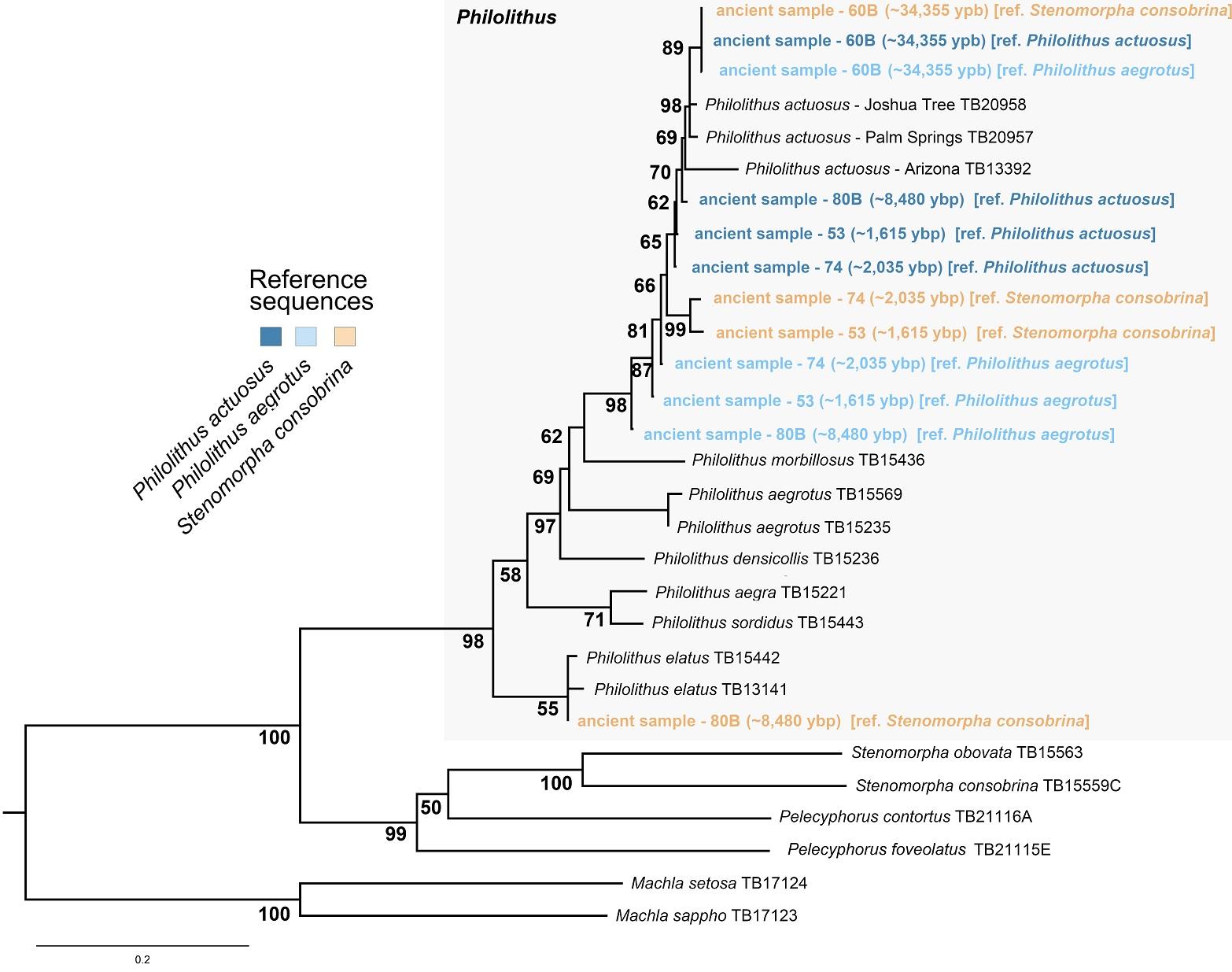

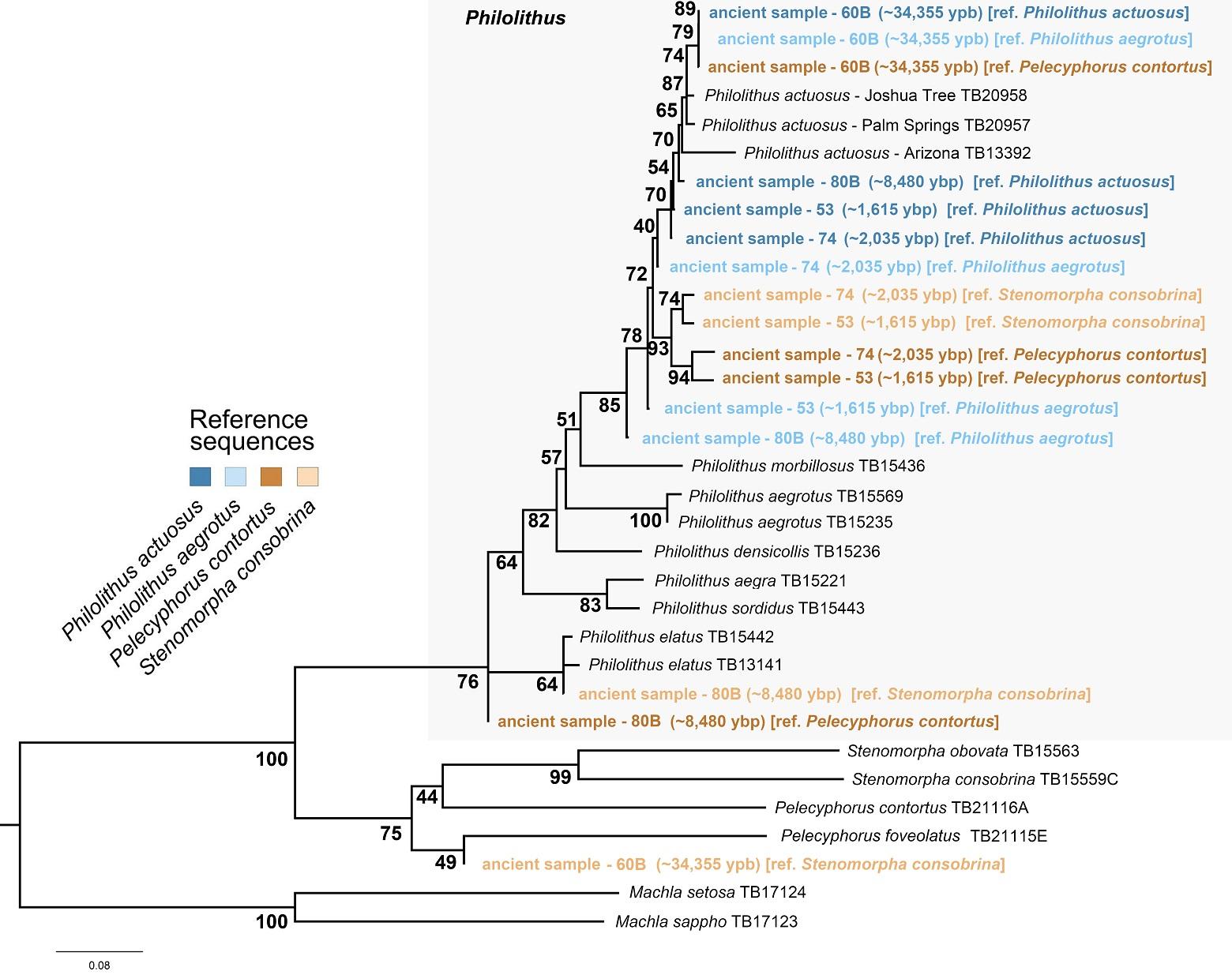

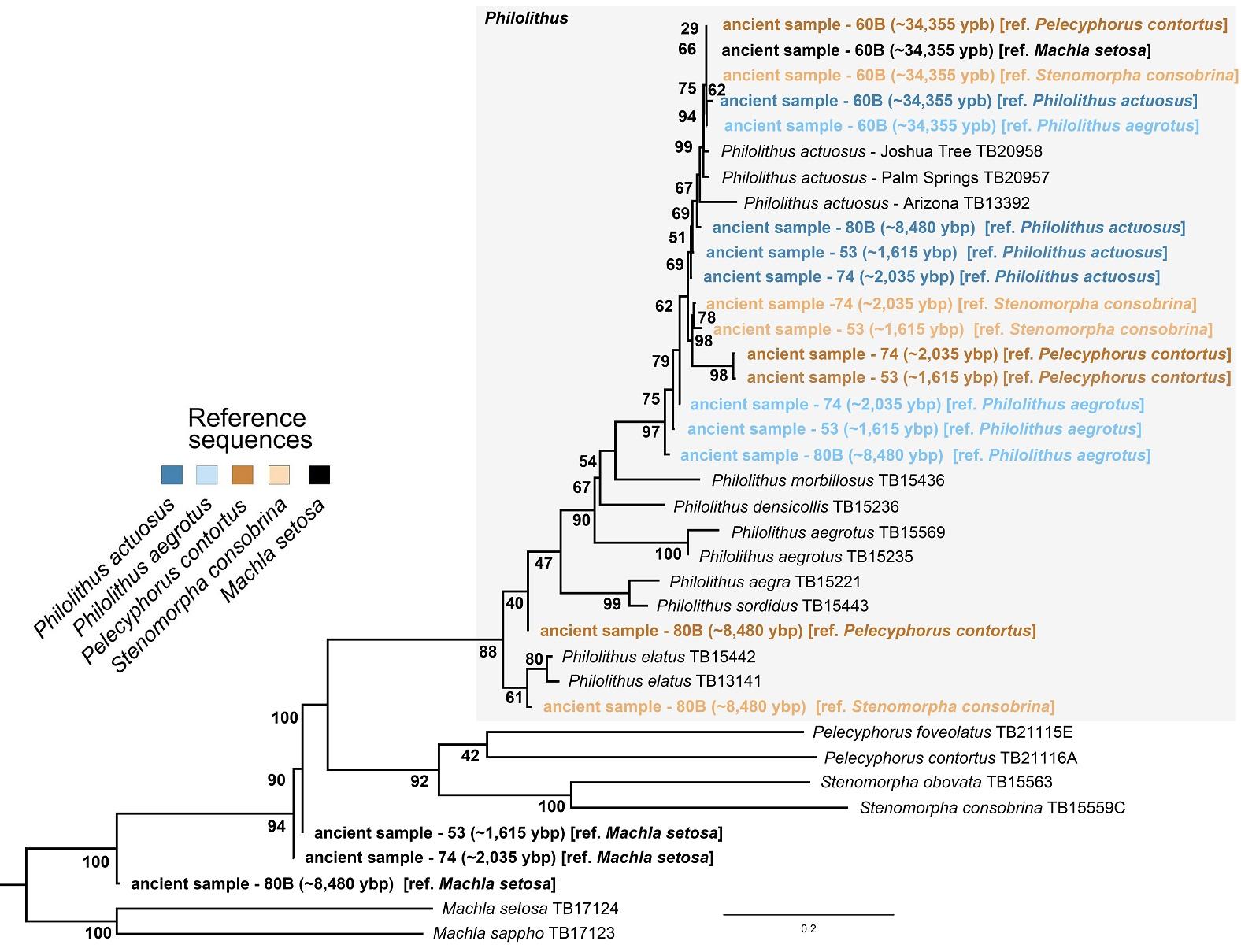

Supplement: Supplementary file 2 — Supplementary Information 2. [file 41598_2021_91896_MOESM2_ESM.docx]
