# Supplementary material for: Recovery and analysis of ancient beetle DNA from subfossil packrat middens using high-throughput sequencing
Source: Sci Rep. 2021 Jun 16;11:12635. doi: 10.1038/s41598-021-91896-8 (PMC8209150; doi:10.1038/s41598-021-91896-8)
Supplement: Supplementary file 6 — Supplementary Information 6. [file 41598_2021_91896_MOESM6_ESM.zip › results_IC44.Phact_JT.filter/Stats_out_MCMC_trace.pdf]

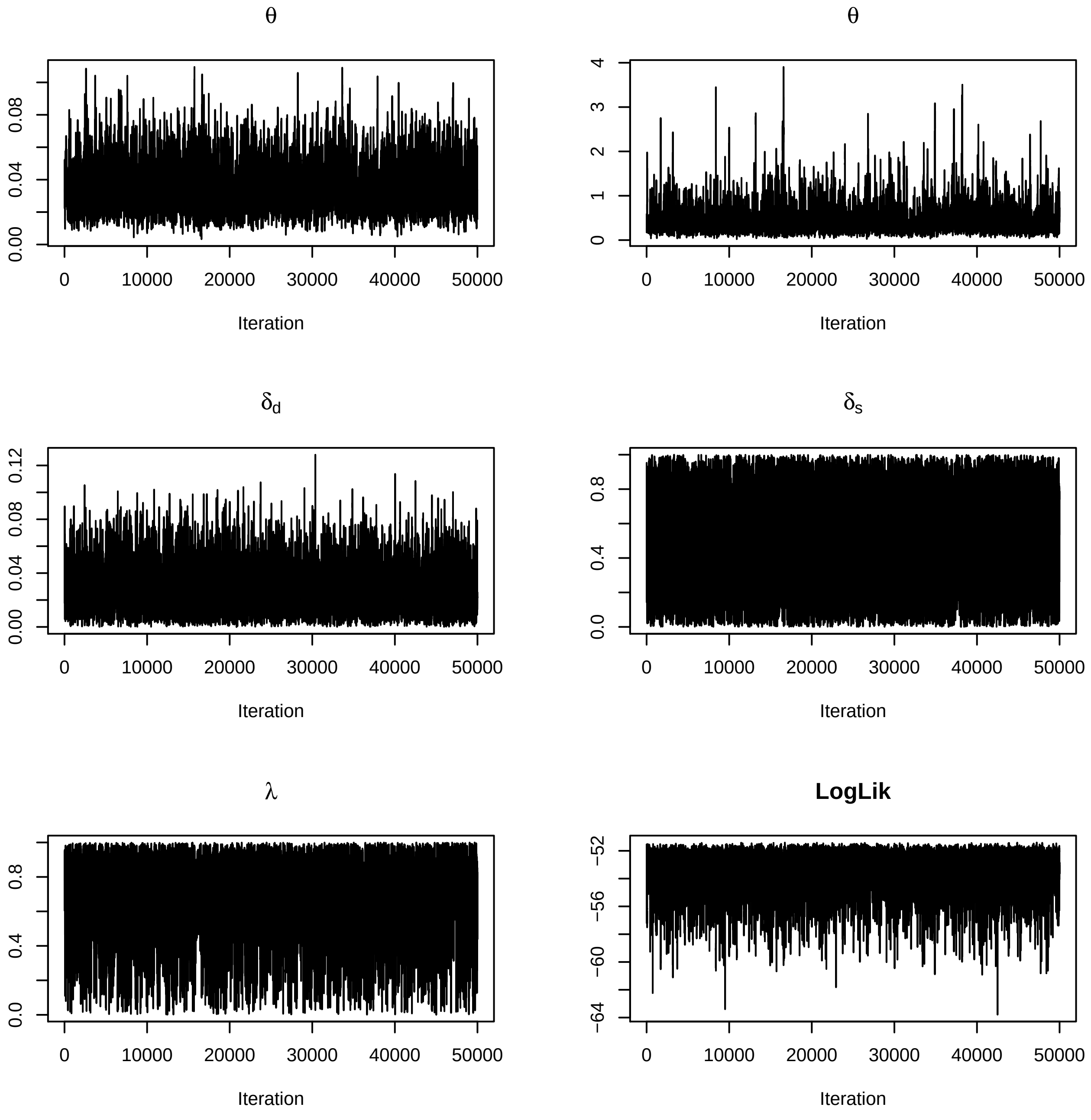
θ
0.00
0.04
0.08
θ
0
1
2
3
4
δd
0.00
0.04
0.08
0.12
δs
0.0
0.4
0.8
λ
0.0
0.4
0.8
LogLik
−52
−56
−60
−64
0
10000
20000
30000
40000
50000
Iteration
