# Supplementary figures and images for: Recovery and analysis of ancient beetle DNA from subfossil packrat middens using high-throughput sequencing
Source: Sci Rep. 2021 Jun 16;11:12635. doi: 10.1038/s41598-021-91896-8 (PMC8209150; doi:10.1038/s41598-021-91896-8)

# IC44.Phact\_JT.filter

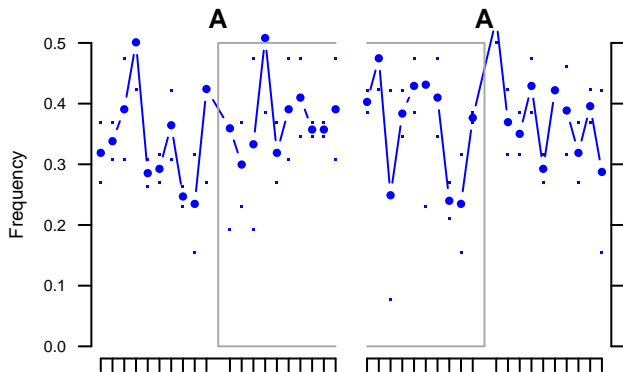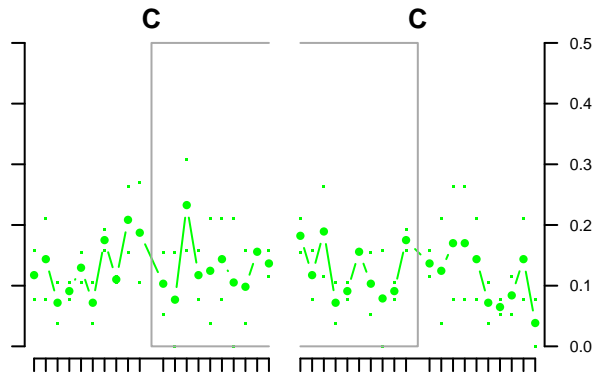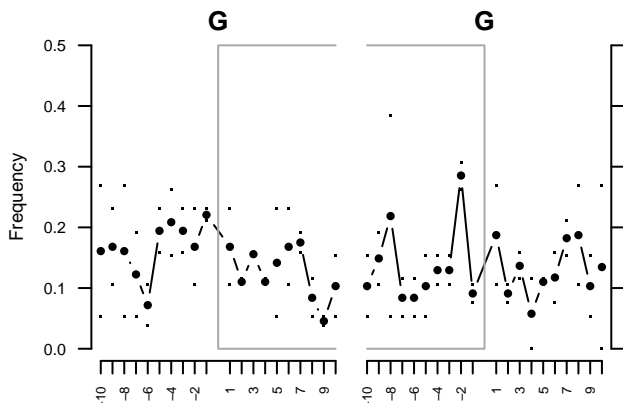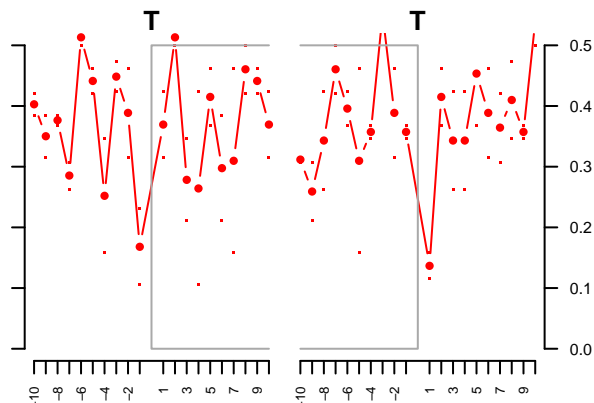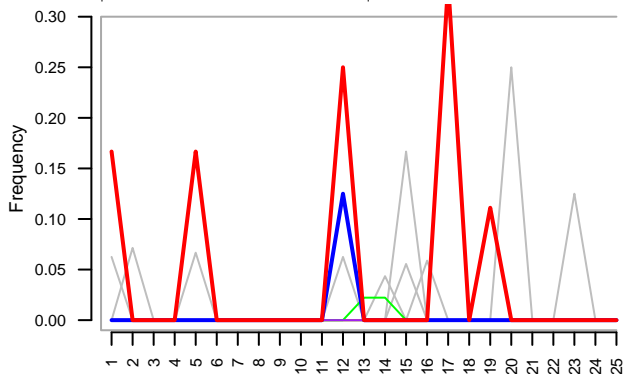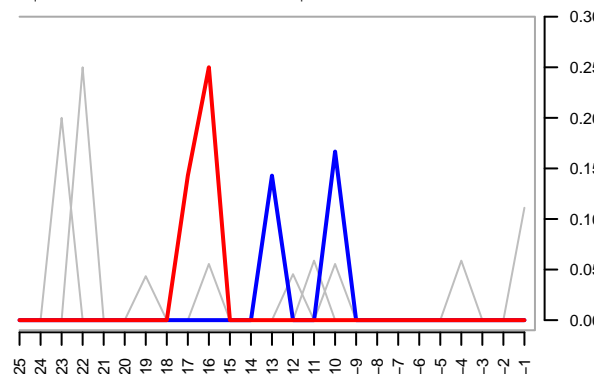

Supplement: Supplementary file 6 — Supplementary Information 6. [file 41598_2021_91896_MOESM6_ESM.zip › results_IC44.Phact_JT.filter/Fragmisincorporation_plot.pdf]

# IC44.Phact\_JT.filter

## Single-end read length distribution

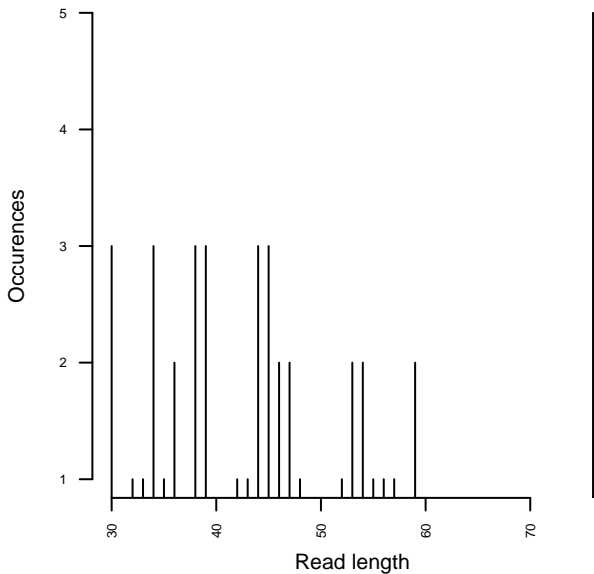

## Single-end read length per strand

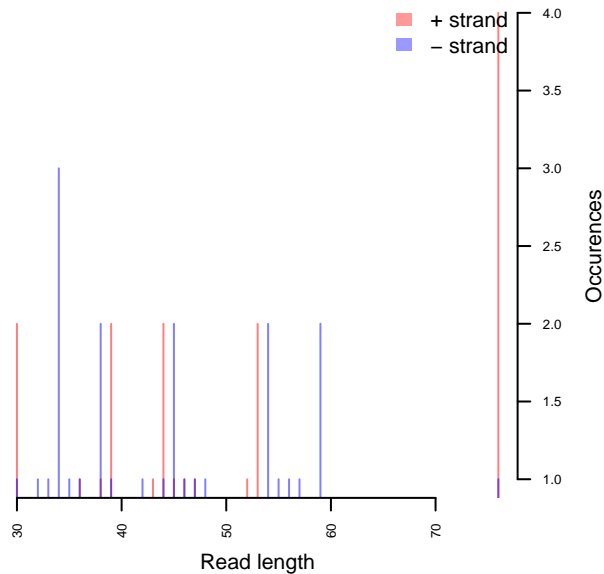

## C>T

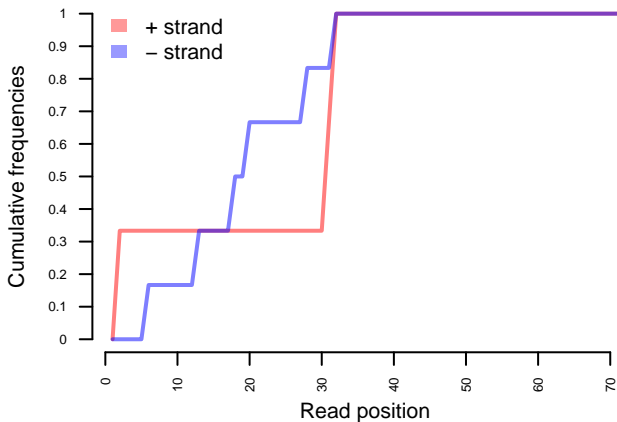

## G>A

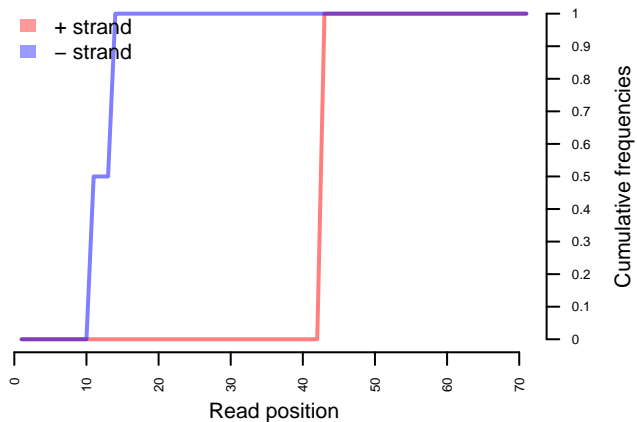

Supplement: Supplementary file 6 — Supplementary Information 6. [file 41598_2021_91896_MOESM6_ESM.zip › results_IC44.Phact_JT.filter/Length_plot.pdf]

$\theta$ 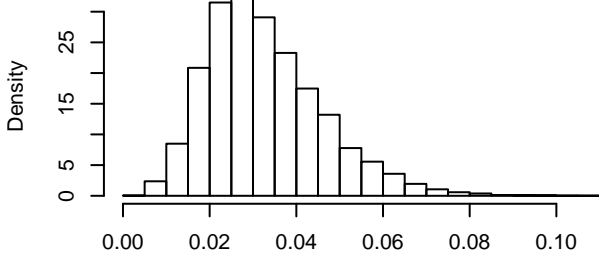 $\rho$ 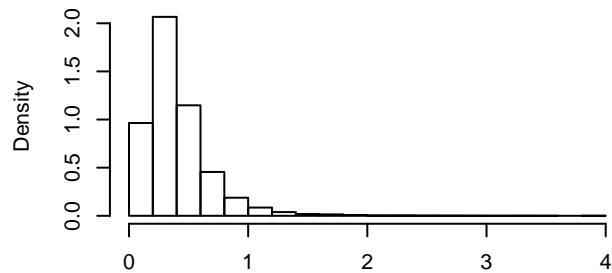 $\delta_d$ 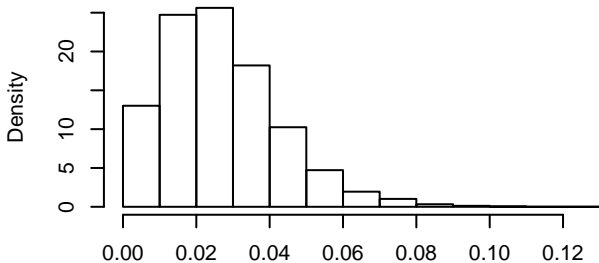 $\delta_s$ 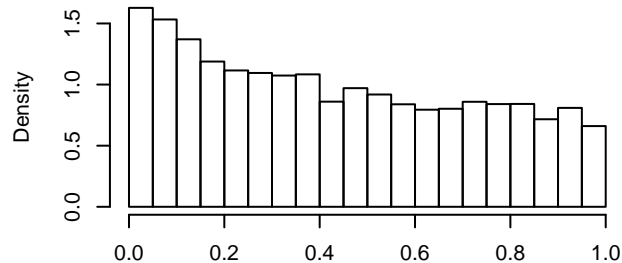 $\lambda$ 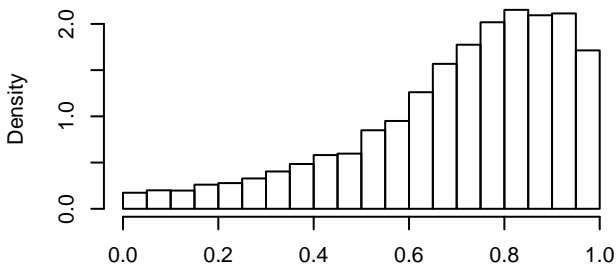**LogLik**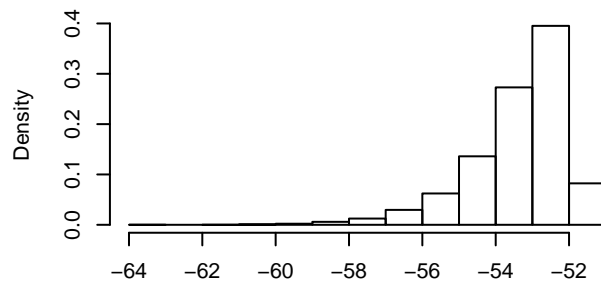

Supplement: Supplementary file 6 — Supplementary Information 6. [file 41598_2021_91896_MOESM6_ESM.zip › results_IC44.Phact_JT.filter/Stats_out_MCMC_hist.pdf]

# Posterior prediction intervals

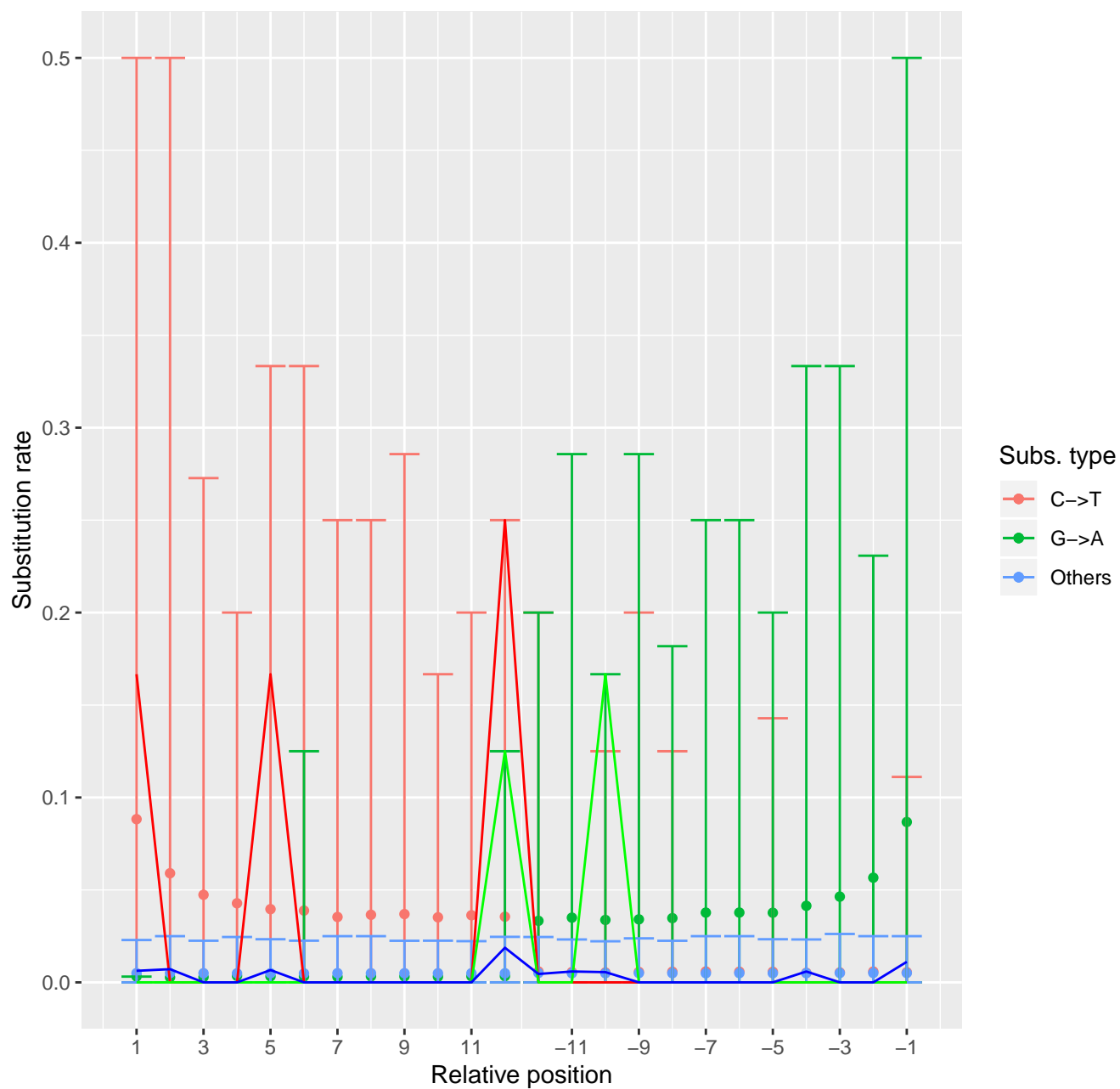

Supplement: Supplementary file 6 — Supplementary Information 6. [file 41598_2021_91896_MOESM6_ESM.zip › results_IC44.Phact_JT.filter/Stats_out_MCMC_post_pred.pdf]

# JR53.Phact\_JT.filter

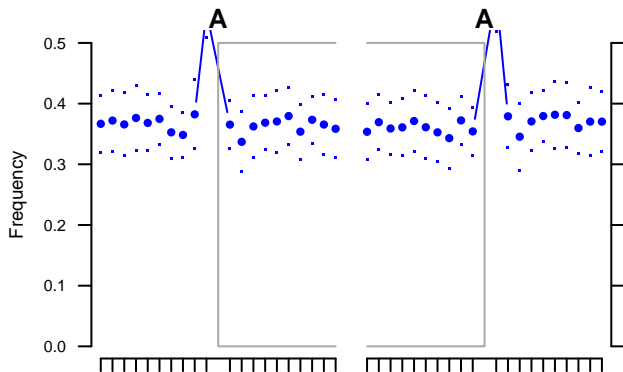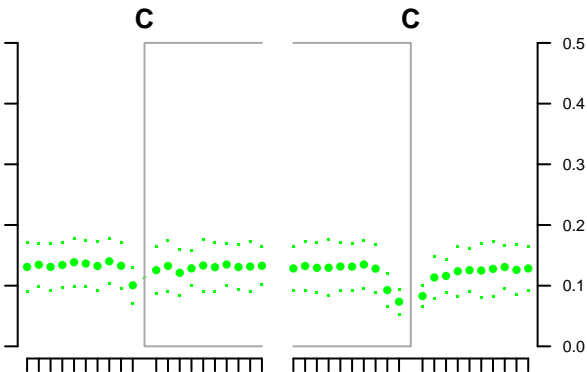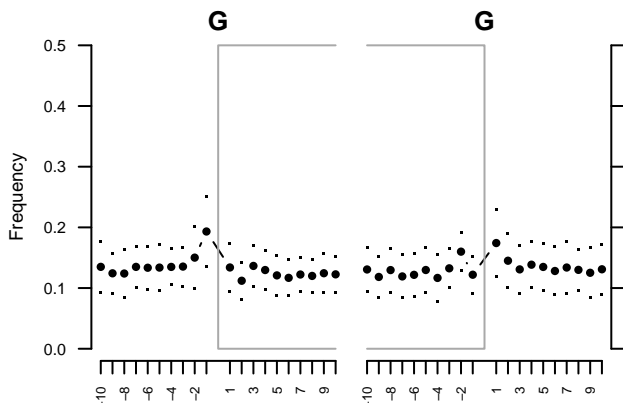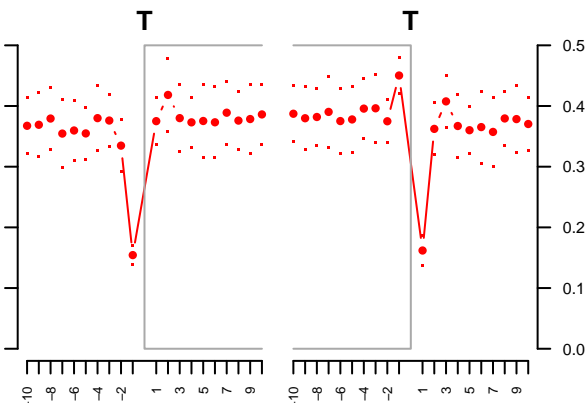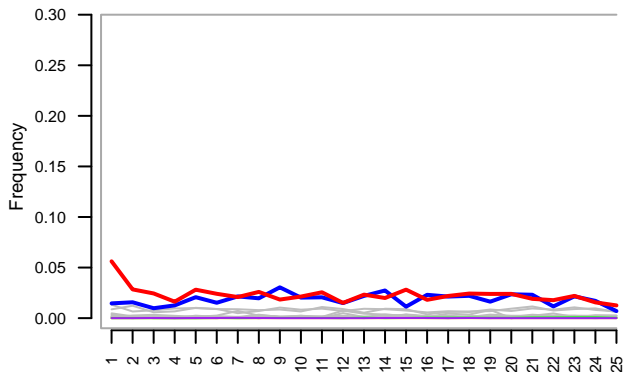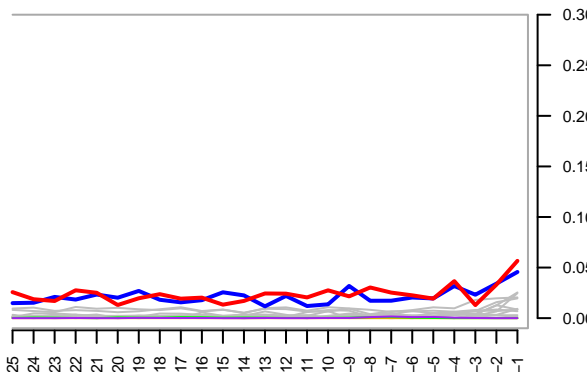

Supplement: Supplementary file 6 — Supplementary Information 6. [file 41598_2021_91896_MOESM6_ESM.zip › results_JR53.Phact_JT.filter/Fragmisincorporation_plot.pdf]

# JR53.Phact\_JT.filter

## Single-end read length distribution

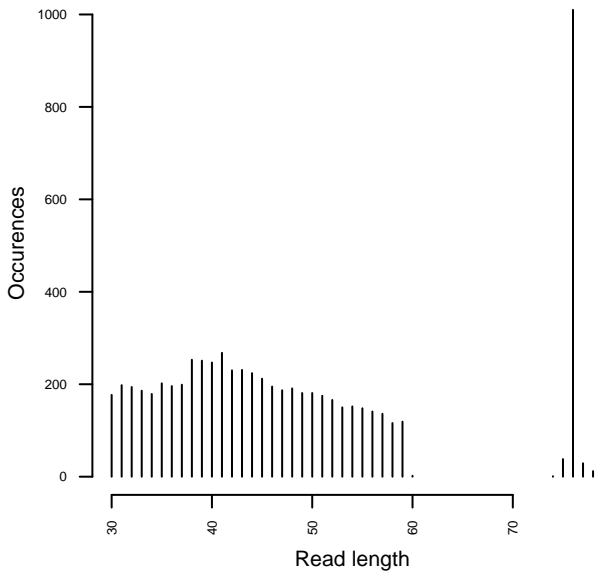

## Single-end read length per strand

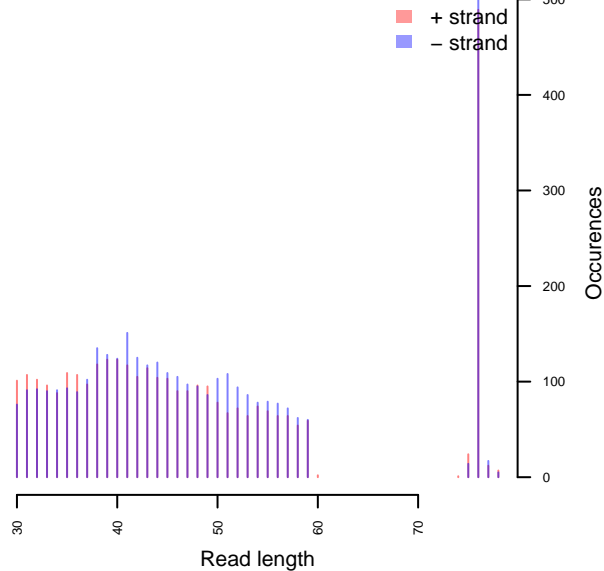

## C>T

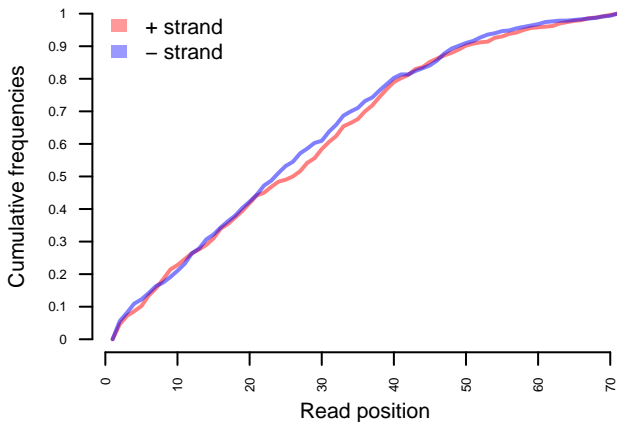

## G>A

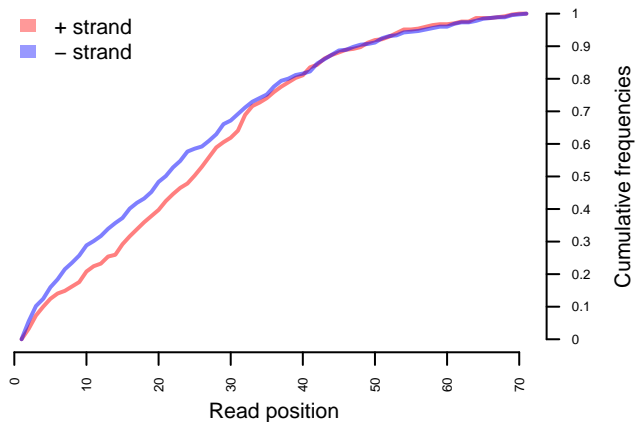

Supplement: Supplementary file 6 — Supplementary Information 6. [file 41598_2021_91896_MOESM6_ESM.zip › results_JR53.Phact_JT.filter/Length_plot.pdf]

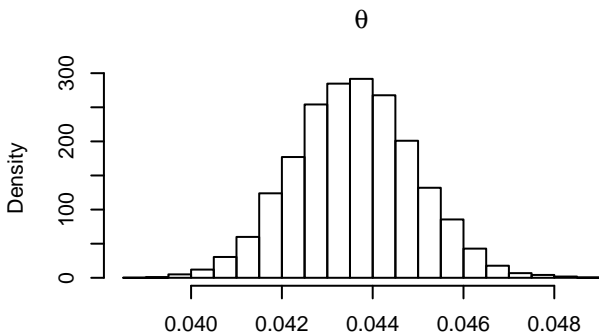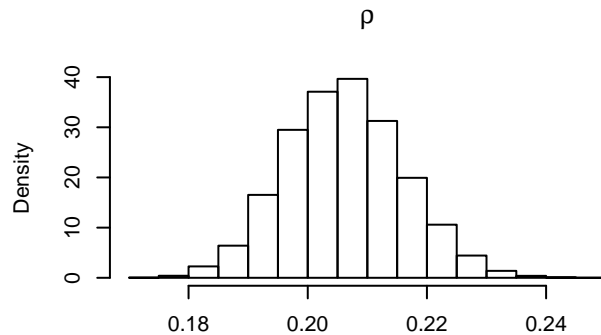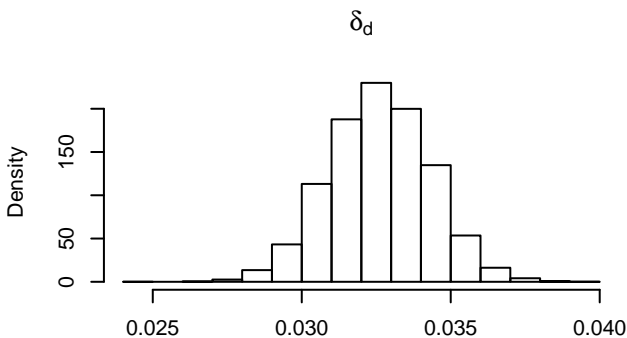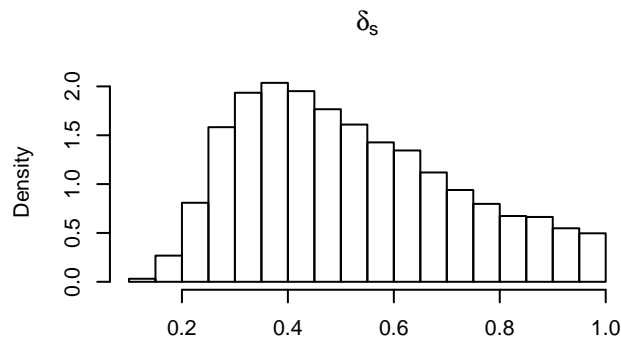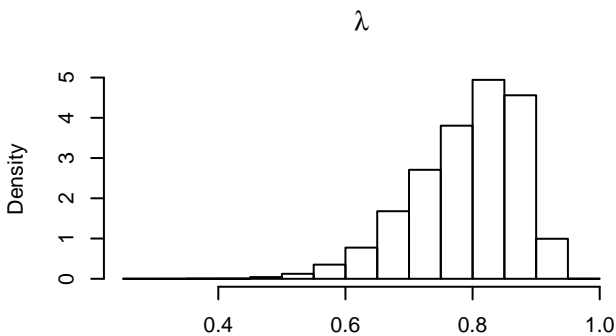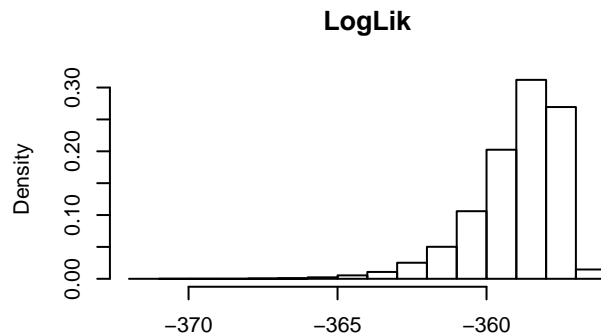

Supplement: Supplementary file 6 — Supplementary Information 6. [file 41598_2021_91896_MOESM6_ESM.zip › results_JR53.Phact_JT.filter/Stats_out_MCMC_hist.pdf]

Posterior prediction intervals

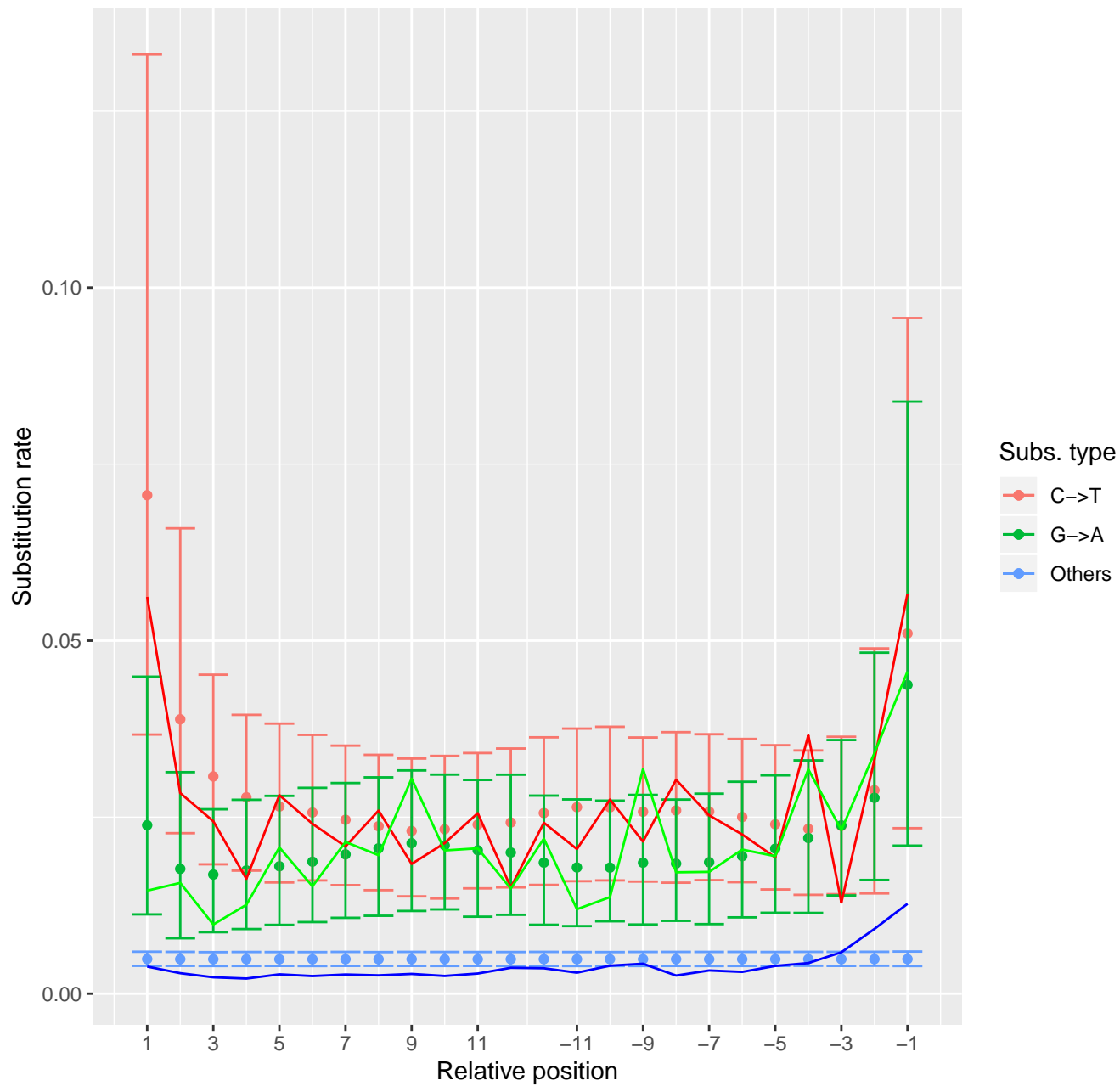

Supplement: Supplementary file 6 — Supplementary Information 6. [file 41598_2021_91896_MOESM6_ESM.zip › results_JR53.Phact_JT.filter/Stats_out_MCMC_post_pred.pdf]

$\theta$ 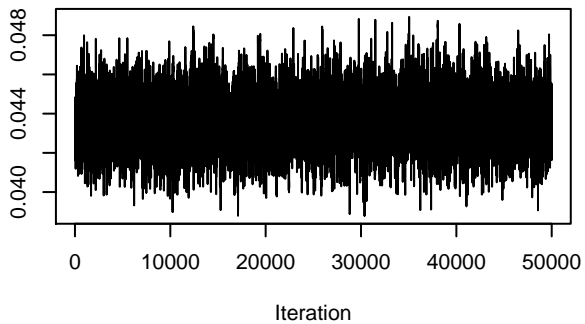 $\theta$ 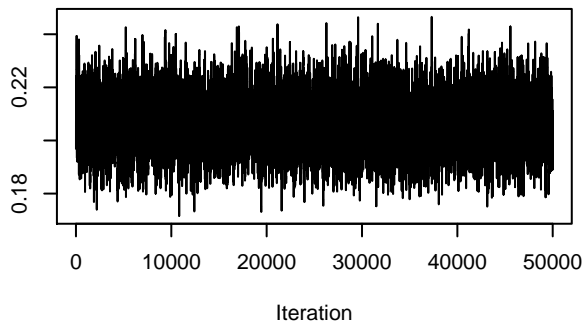 $\delta_d$ 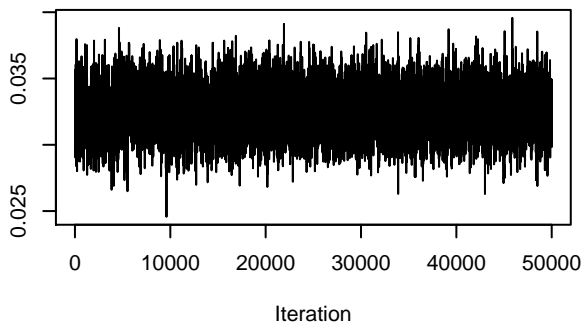 $\delta_s$ 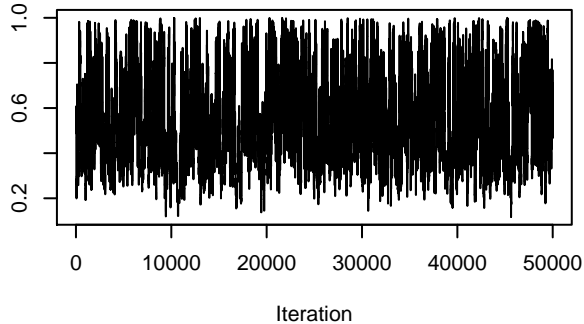 $\lambda$ 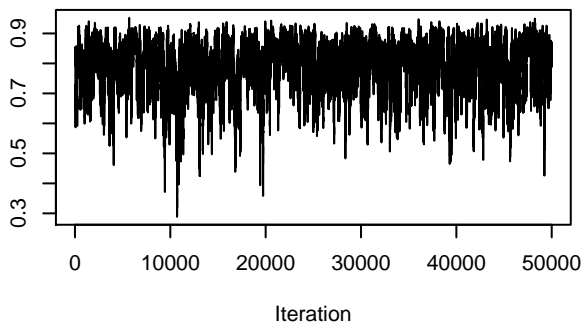**LogLik**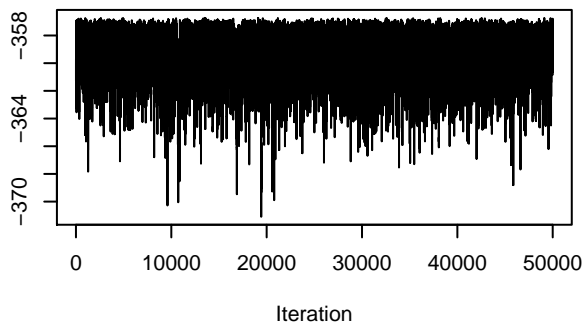

Supplement: Supplementary file 6 — Supplementary Information 6. [file 41598_2021_91896_MOESM6_ESM.zip › results_JR53.Phact_JT.filter/Stats_out_MCMC_trace.pdf]

# JT2.Phact\_JT.filter

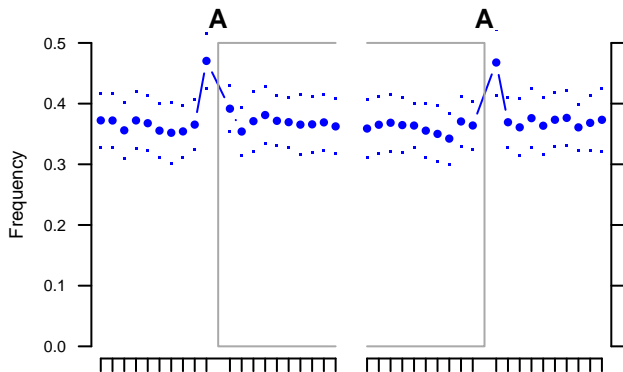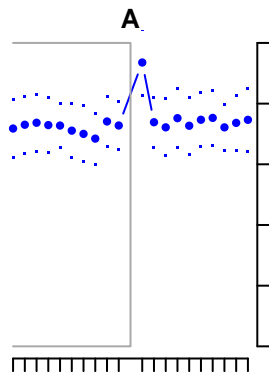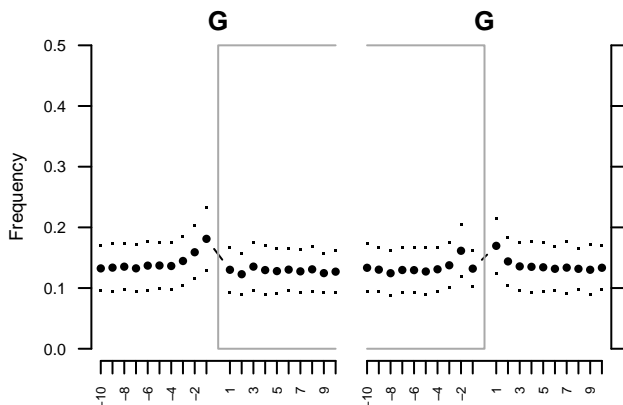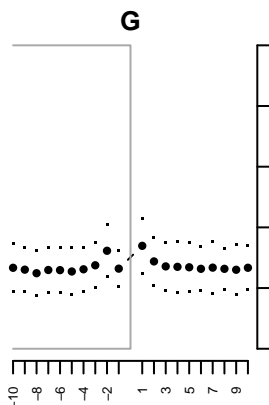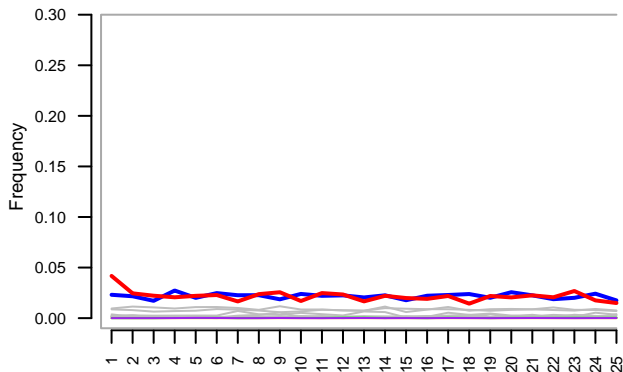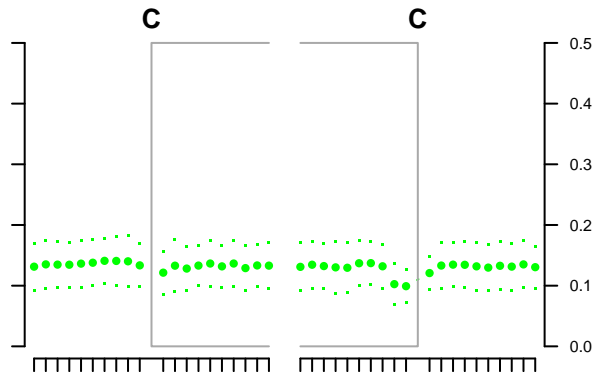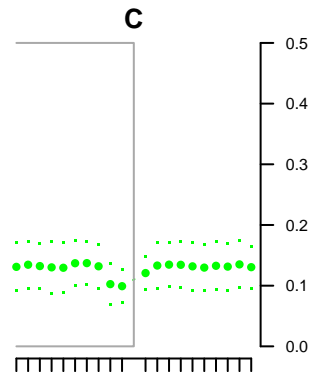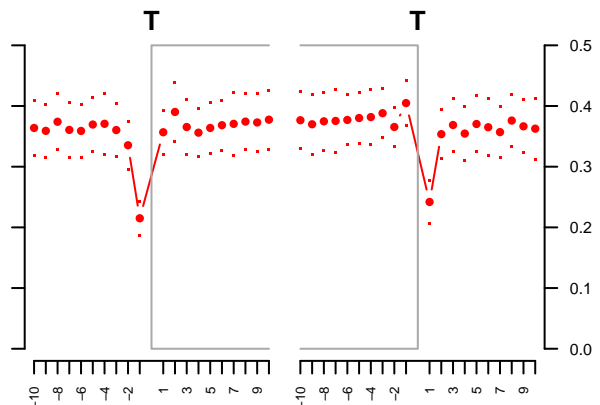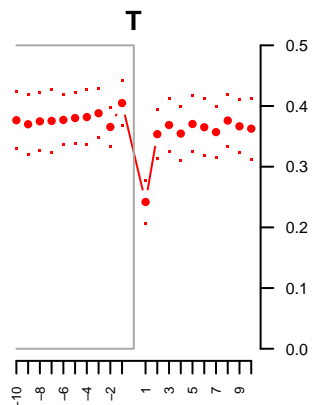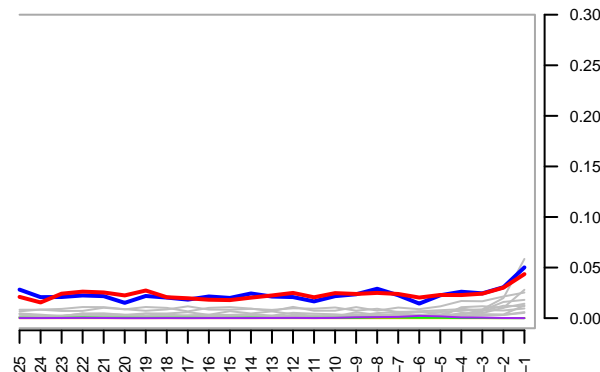

Supplement: Supplementary file 6 — Supplementary Information 6. [file 41598_2021_91896_MOESM6_ESM.zip › results_JT2.Phact_JT.filter/Fragmisincorporation_plot.pdf]

# JT2.Phact\_JT.filter

## Single-end read length distribution

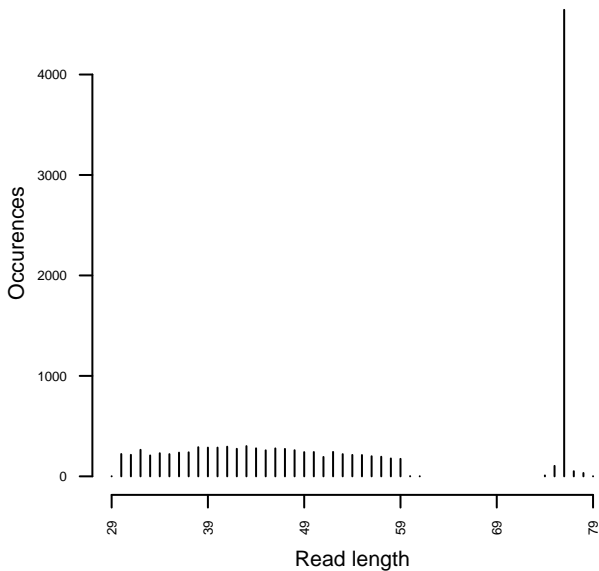

## Single-end read length per strand

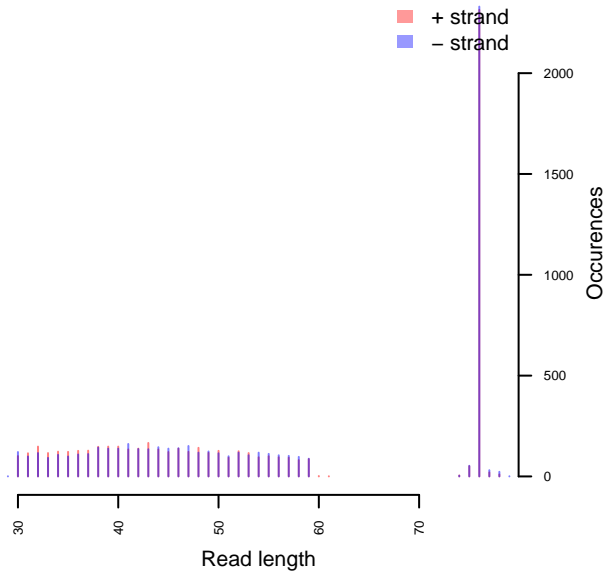

## C>T

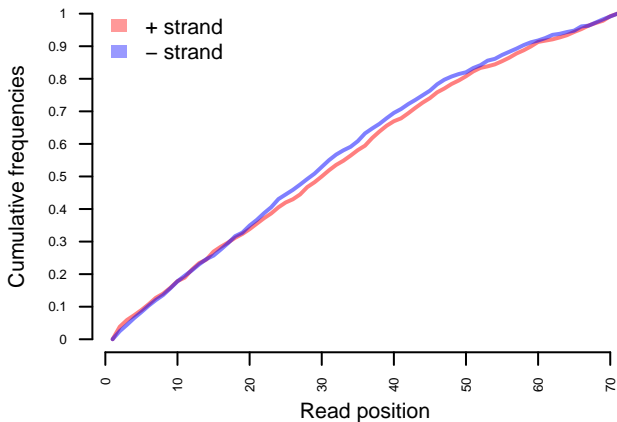

## G>A

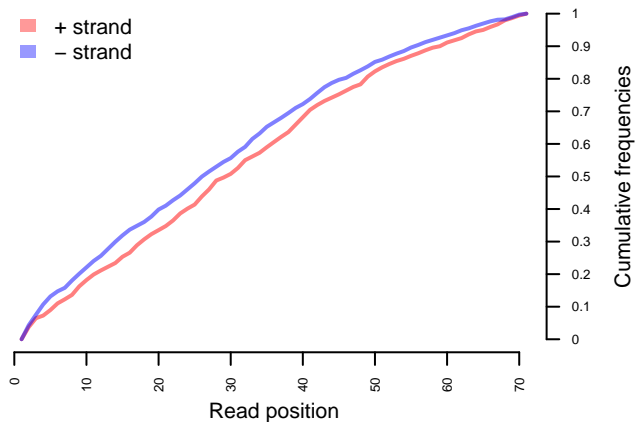

Supplement: Supplementary file 6 — Supplementary Information 6. [file 41598_2021_91896_MOESM6_ESM.zip › results_JT2.Phact_JT.filter/Length_plot.pdf]

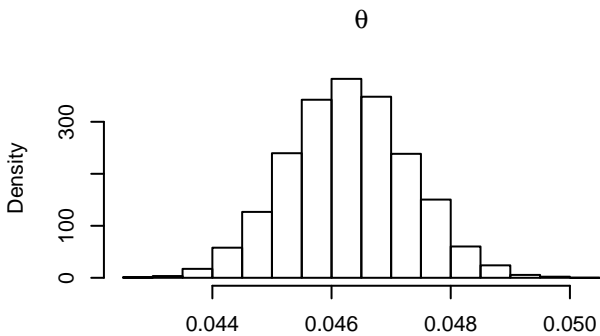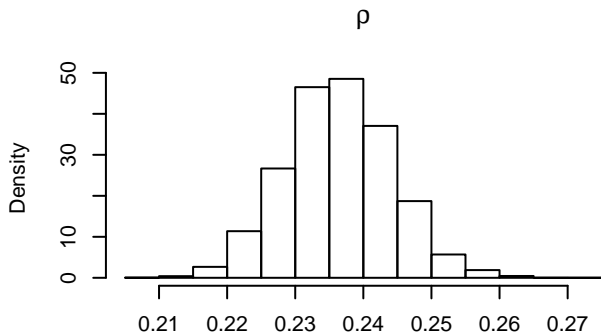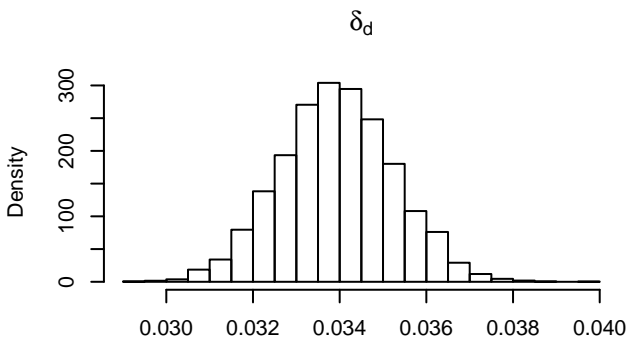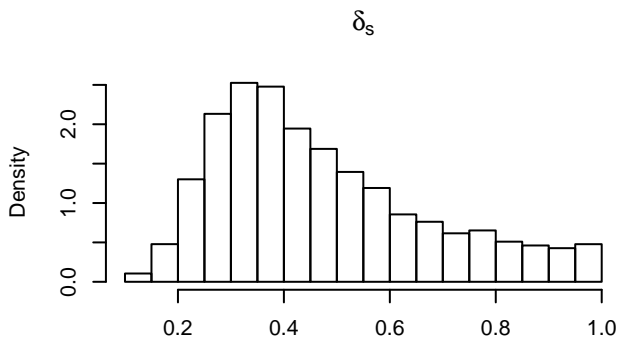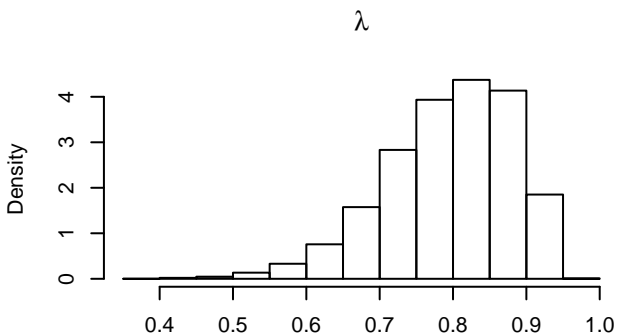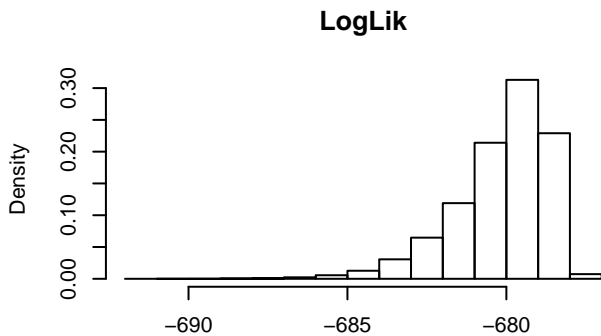

Supplement: Supplementary file 6 — Supplementary Information 6. [file 41598_2021_91896_MOESM6_ESM.zip › results_JT2.Phact_JT.filter/Stats_out_MCMC_hist.pdf]

Posterior prediction intervals

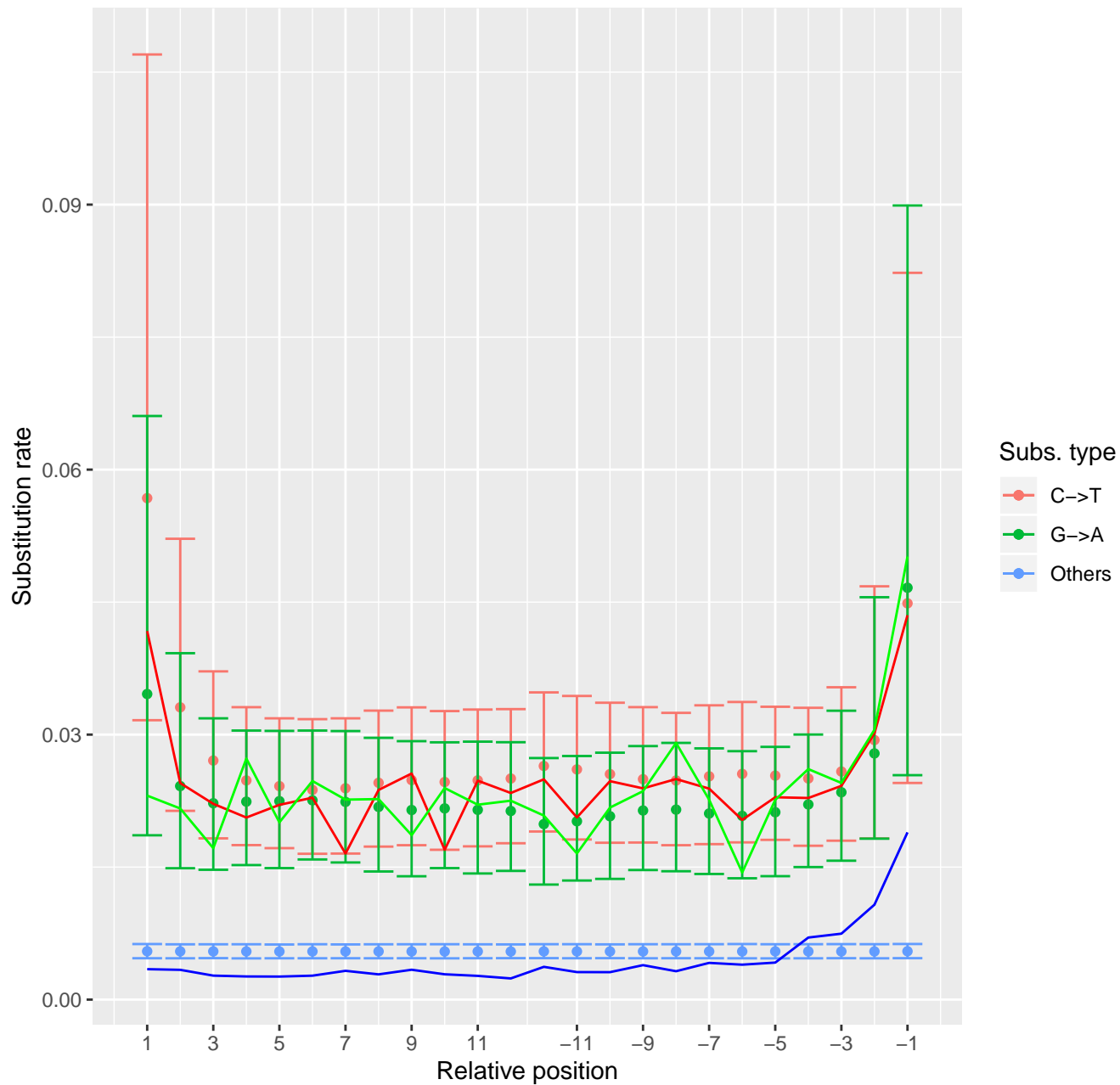

Supplement: Supplementary file 6 — Supplementary Information 6. [file 41598_2021_91896_MOESM6_ESM.zip › results_JT2.Phact_JT.filter/Stats_out_MCMC_post_pred.pdf]

$\theta$ 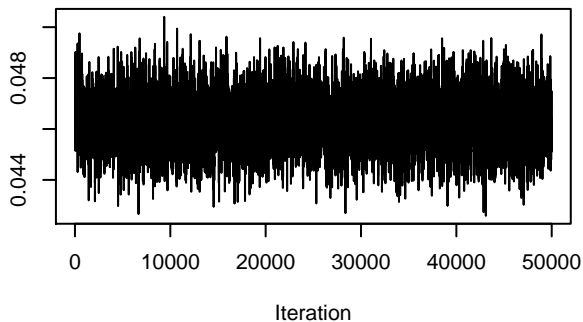 $\theta$ 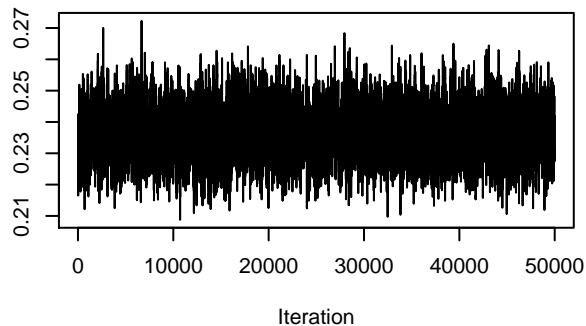 $\delta_d$ 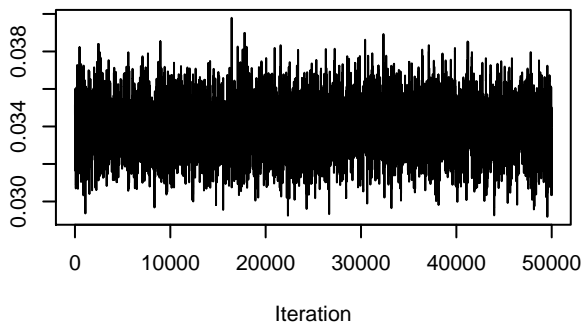 $\delta_s$ 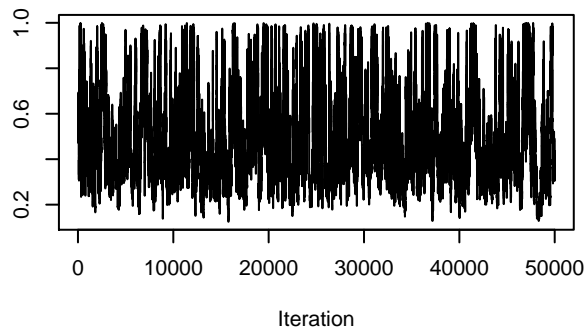 $\lambda$ 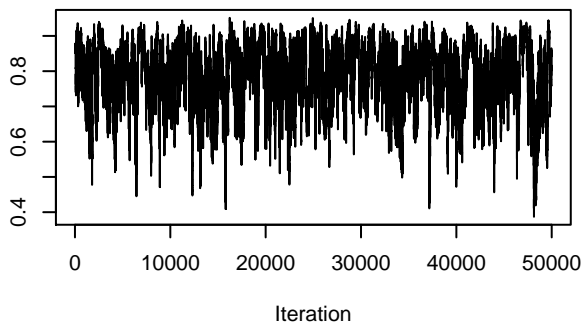**LogLik**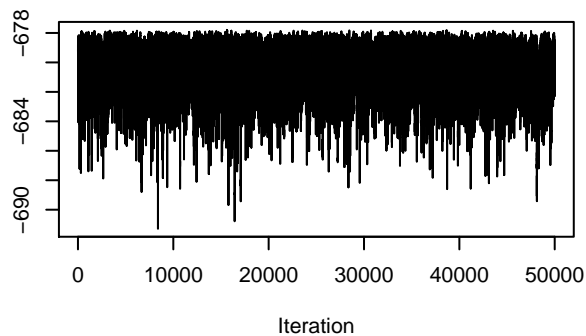

Supplement: Supplementary file 6 — Supplementary Information 6. [file 41598_2021_91896_MOESM6_ESM.zip › results_JT2.Phact_JT.filter/Stats_out_MCMC_trace.pdf]

# JT8.Phact\_JT.filter

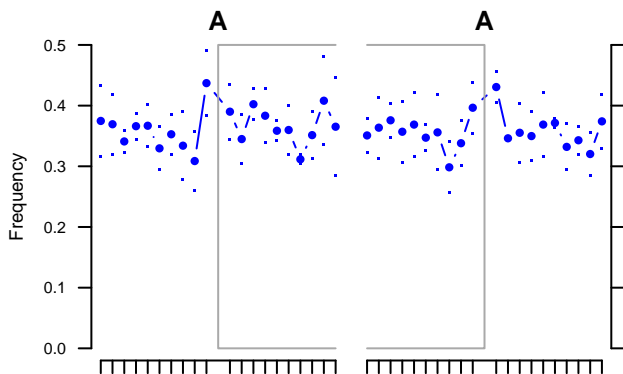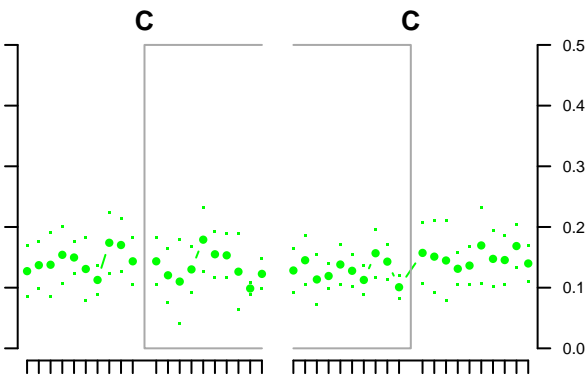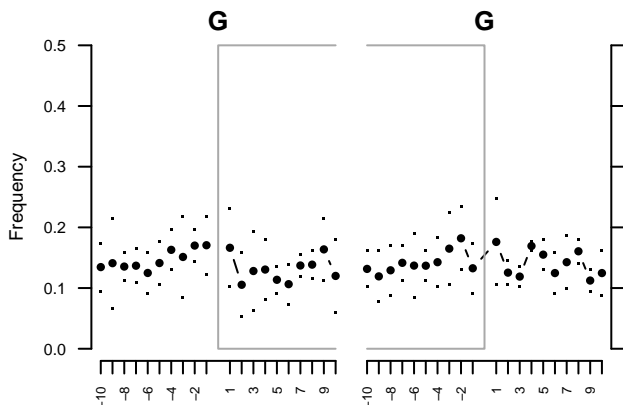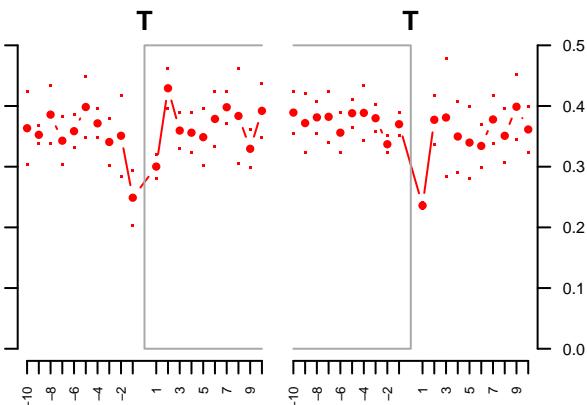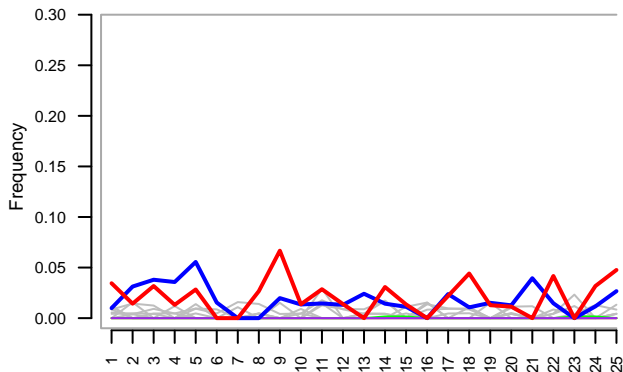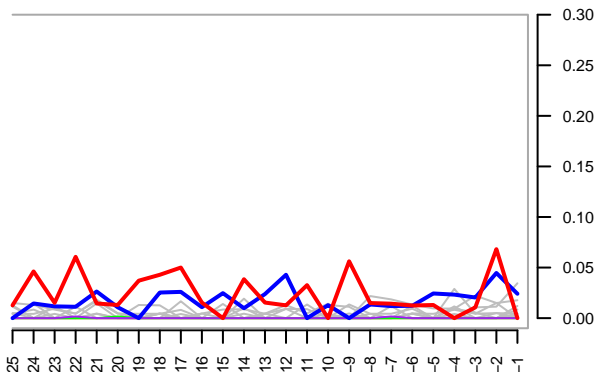

Supplement: Supplementary file 6 — Supplementary Information 6. [file 41598_2021_91896_MOESM6_ESM.zip › results_JT8.Phact_JT.filter/Fragmisincorporation_plot.pdf]

# JT8.Phact\_JT.filter

## Single-end read length distribution

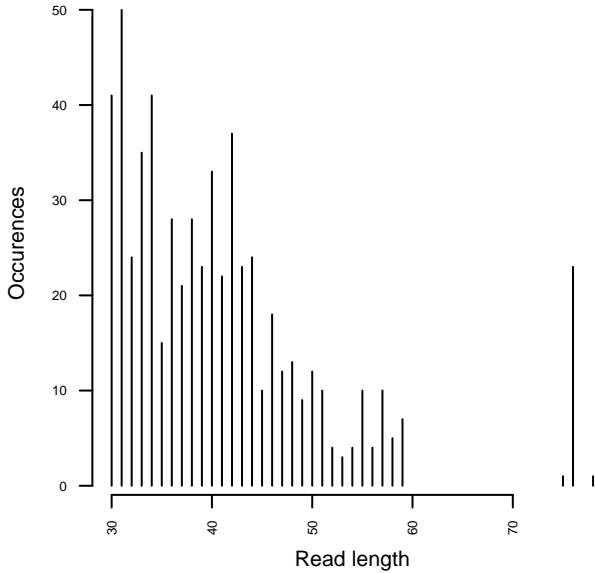

## Single-end read length per strand

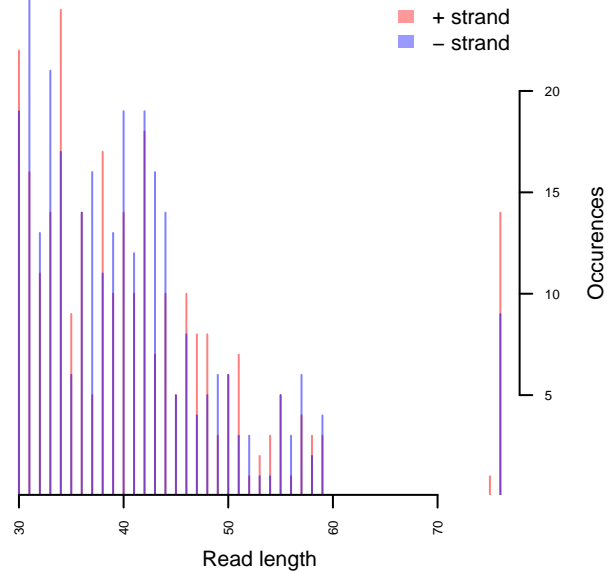

## C>T

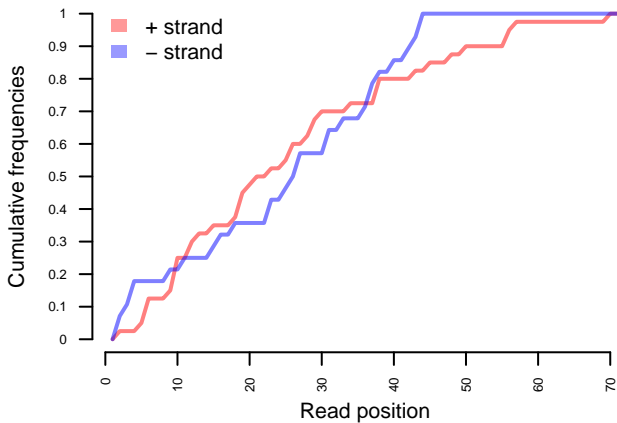

## G>A

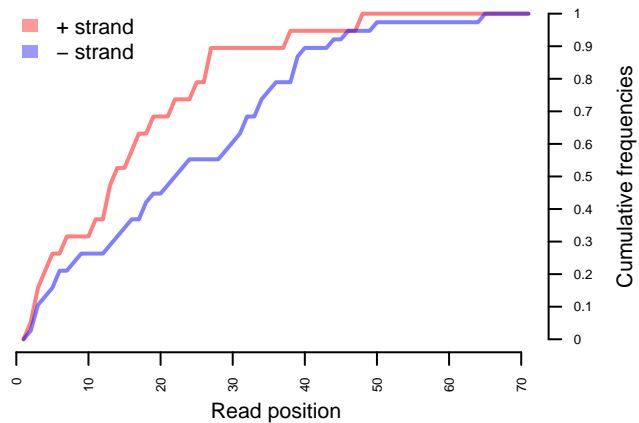

Supplement: Supplementary file 6 — Supplementary Information 6. [file 41598_2021_91896_MOESM6_ESM.zip › results_JT8.Phact_JT.filter/Length_plot.pdf]

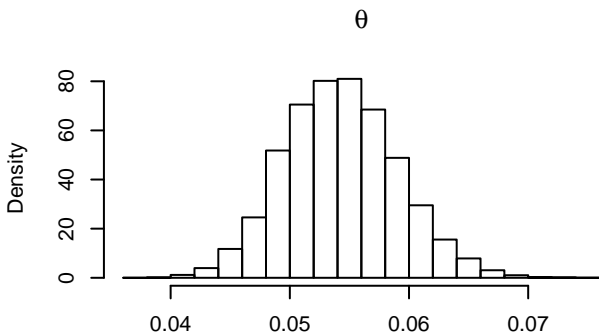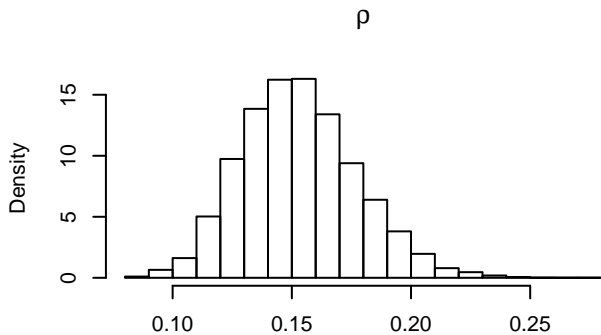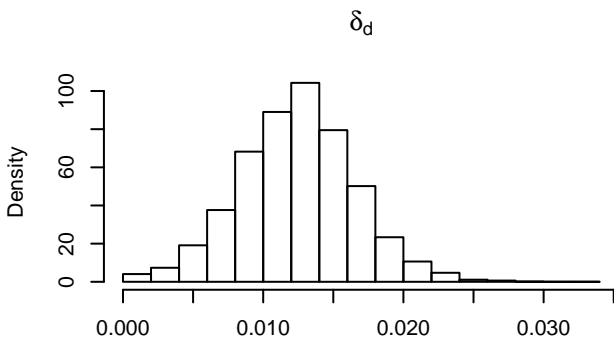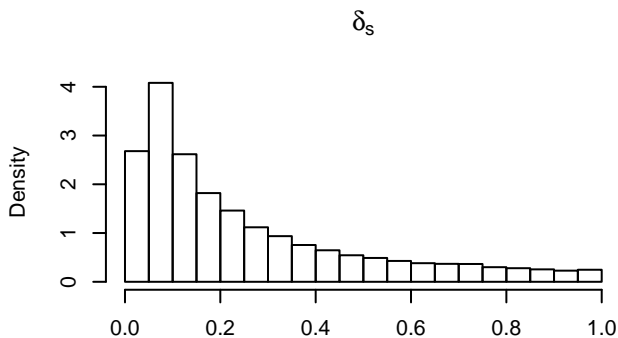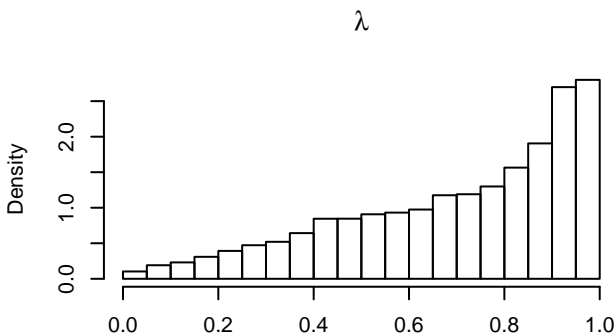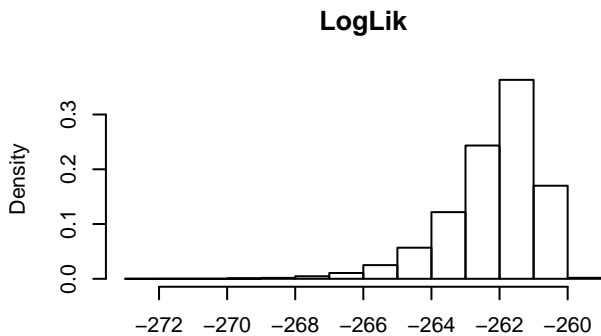

Supplement: Supplementary file 6 — Supplementary Information 6. [file 41598_2021_91896_MOESM6_ESM.zip › results_JT8.Phact_JT.filter/Stats_out_MCMC_hist.pdf]

# Posterior prediction intervals

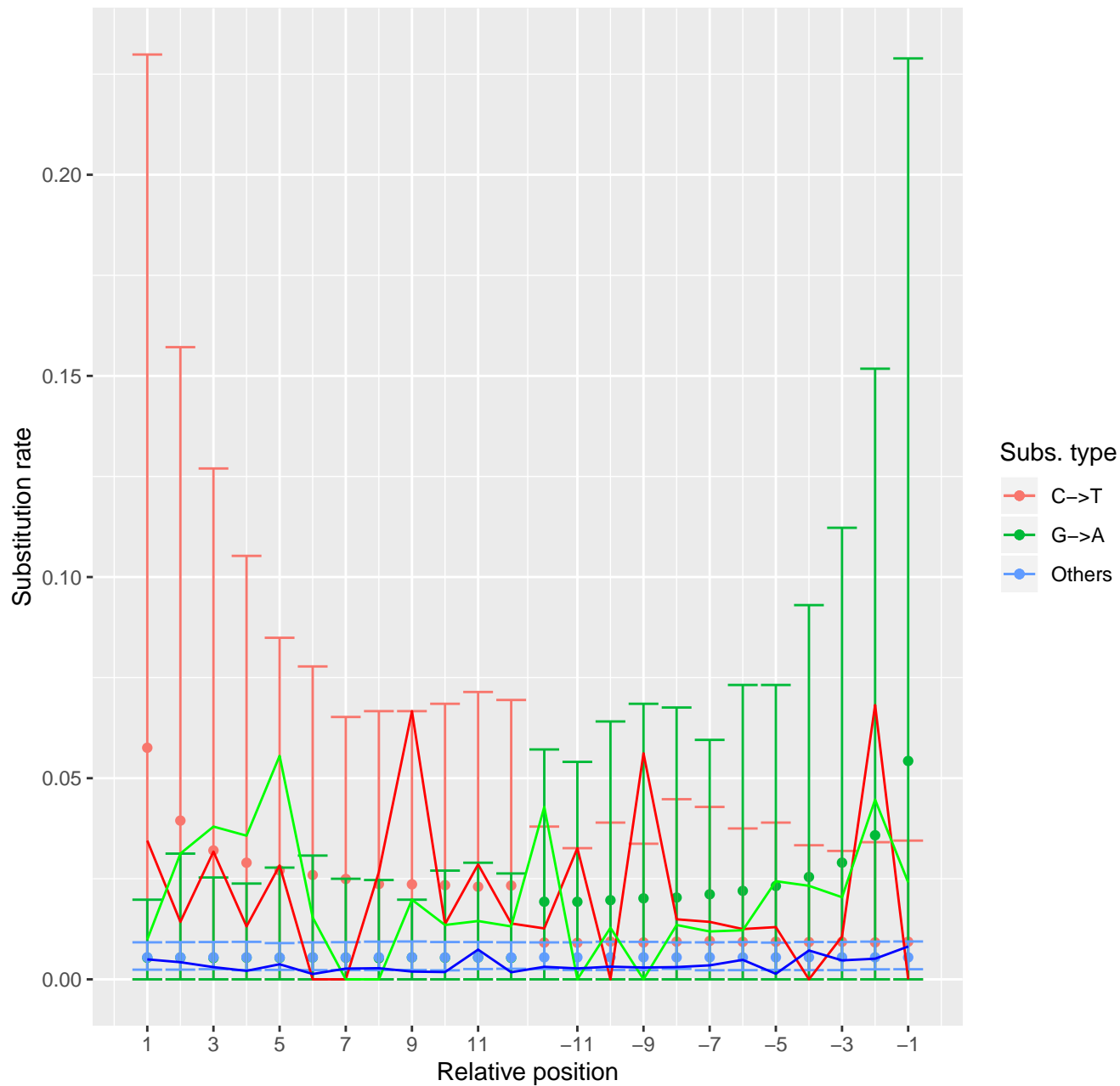

Supplement: Supplementary file 6 — Supplementary Information 6. [file 41598_2021_91896_MOESM6_ESM.zip › results_JT8.Phact_JT.filter/Stats_out_MCMC_post_pred.pdf]

$\theta$ 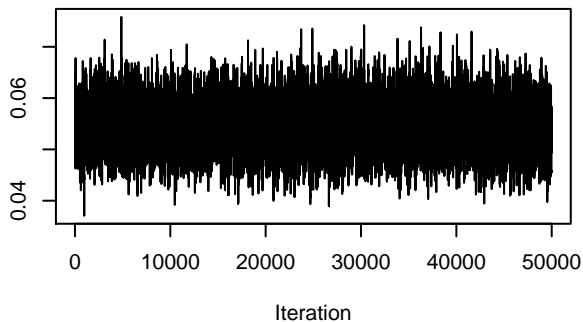 $\theta$ 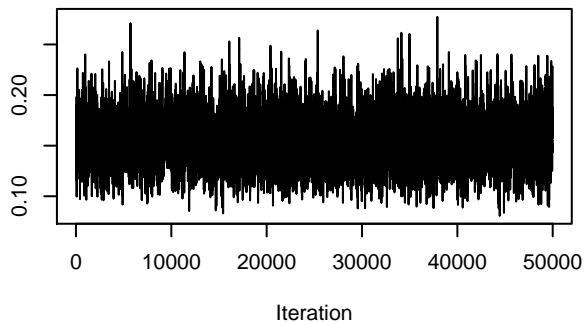 $\delta_d$ 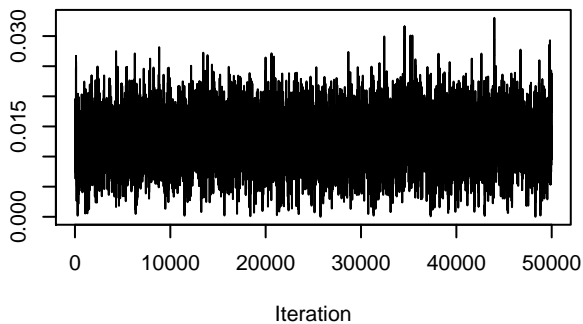 $\delta_s$ 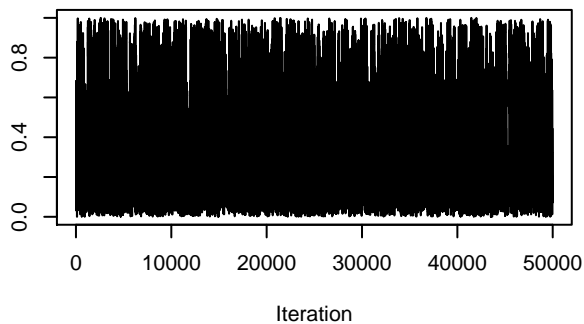 $\lambda$ 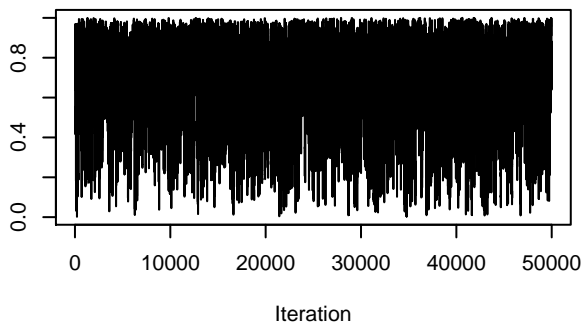**LogLik**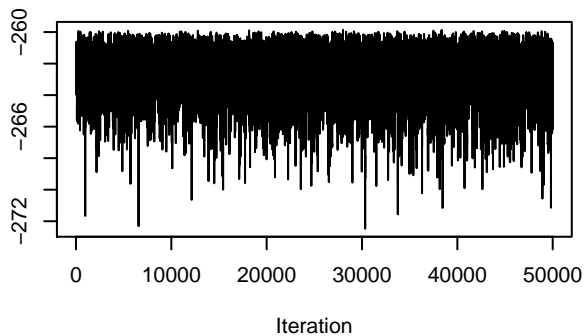

Supplement: Supplementary file 6 — Supplementary Information 6. [file 41598_2021_91896_MOESM6_ESM.zip › results_JT8.Phact_JT.filter/Stats_out_MCMC_trace.pdf]
